# Numerical simulation of heat transfer performance of spiral wound heat exchanger under sloshing condition

**DOI:** 10.1371/journal.pone.0295315

**Published:** 2023-12-11

**Authors:** Longfei Dong, Congcong Dong, Xiao Wu

**Affiliations:** 1 Shandong Institute of Petroleum and Chemical Technology, Dongying, Shandong, China; 2 Dongying Experimental Middle School, Dongying, Shandong, China; Southwest Jiaotong University, CHINA

## Abstract

LNG floating production storage and offloading (FPSO) unit is a new type of floating production unit developed for offshore natural gas fields. It performs the functions of natural gas extraction, pretreatment, liquefaction, and storage. In this study, the heat transfer characteristics of a spiral-wound heat exchanger were studied using numerical simulations, which provides a basis for equipment selection and structural optimization of spiral-wound heat exchangers. The main research contents and conclusions are as follows. Under land-based simulation conditions, the heat transfer coefficient of the shell side of the spiral-wound heat exchanger decreases with an increase in the winding angle of the heat exchange tube, decreases with an increase in the spacing of the heat exchange tube, and increases with an increase in the outer diameter of the heat exchange tube. When the winding angle increased from 5° to 8°, the heat transfer coefficient decreased by 6.70%. The heat transfer coefficient of the shell side decreased by 13.21% when the heat exchange tube spacing increased from 14 to 17 mm. The heat transfer coefficient of the shell side increased by 22.89% when the outer diameter of the heat exchange tube increased from 9 to 12 mm. When the sloshing angle is constant, the heat transfer coefficient of the spiral-wound heat exchanger decreases with an increase in the winding angle and spacing of the heat exchange tubes, and increases with an increase in the outer diameter of the heat exchange tubes. When the structural parameters of the heat exchanger are constant, the heat transfer coefficient decreases as the sloshing angle increases. When the sloshing angle was less than 3°, the sloshing promoted heat transfer on the shell side. When the sloshing angle was higher than 7°, the heat transfer effect of the shell side deteriorated considerably, which weakened the heat transfer performance of the heat exchanger. When the sloshing period is constant, the heat transfer coefficient of the wound heat exchanger decreases with an increase in the winding angle and spacing of the heat exchange tubes, and increases with an increase in the outer diameter of the heat exchange tubes. When the structural parameters of the heat exchanger are constant, the heat transfer coefficient increases with sloshing period. When the sloshing period was greater than 15 s, the influence of sloshing on the heat transfer on the shell side of the heat exchanger was relatively weak.

## 1. Introduction

**Fig 1:**
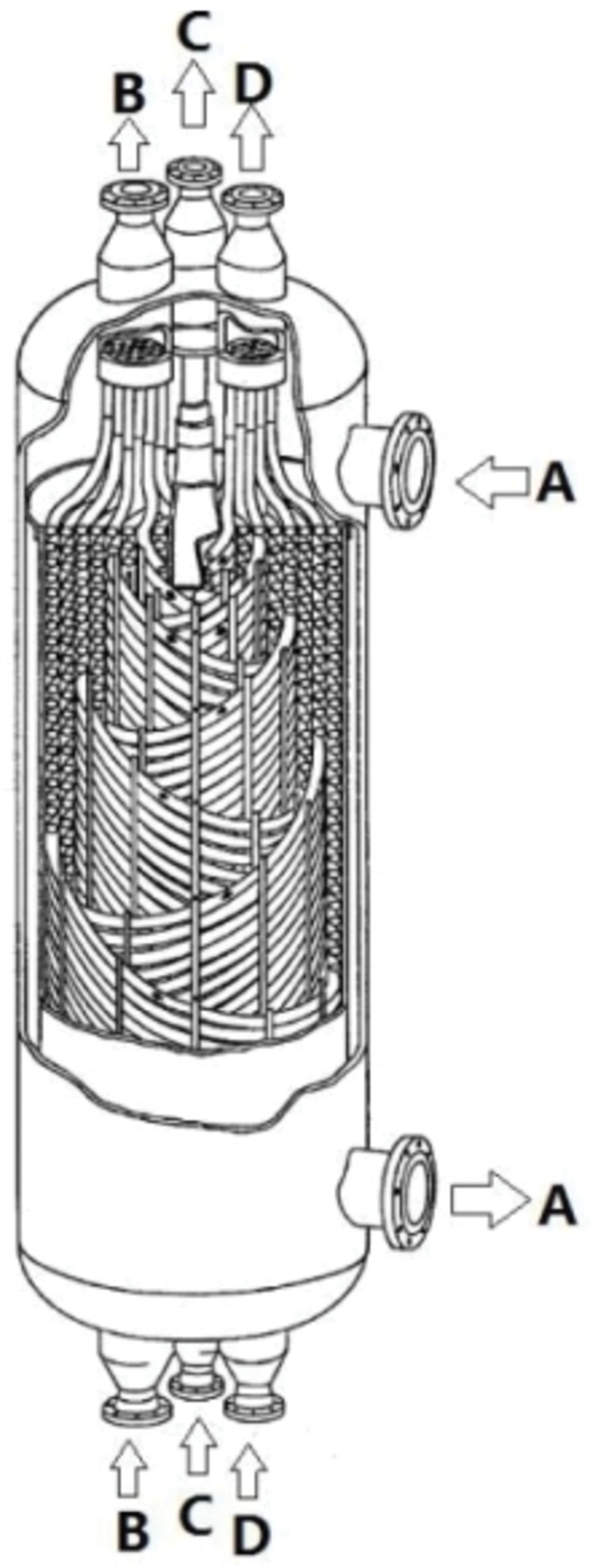
Schematic of the geometric structure of the LNG coil heat exchanger.

With the adjustment of China ’s energy consumption structure, the proportion of natural gas in the energy supply will continue to increase as a representative of clean energy. The South China Sea is rich in oil and gas resources, 70% of which come from deep waters. Currently, the most commonly used offshore natural gas gathering and transportation mode is submarine pipelines. However, owing to its high laying cost, limited pipeline pressure, and pipe diameter, its transportation capacity is limited. Therefore, it is necessary to develop new natural gas collection and transportation modes to solve this problem. LNG floating production storage and offloading (FPSO) unit is a new type of floating production unit for the development of offshore natural gas fields. It performs the functions of natural gas extraction, pretreatment, liquefaction, and storage, and is suitable for the development of deep-sea small- and medium-sized gas fields [1]. Its structure is shown in Fig 1 [2].

Spiral-wound heat exchanger is a type of shell-and-tube heat exchanger. Researchers have conducted experimental and theoretical research on the performance of spiral-wound heat exchangers. Julio et al. [3] introduced a detailed thermo-hydraulic model of a heat exchanger for low-temperature applications, including lumped parameter, distributed parameter, and flow development models. However, the influence of pressure drop and local flow mixing in the heat transfer process was neglected in the model calculation process. Gao et al. [4] conducted experiments on the heat transfer and pressure drop performance of the shell side of a finned coil heat exchanger and computed the shell-side heat transfer and pressure drop correlations. Yin et al. [5] established a heat balance equation for a microsegment of the metal wall of a parallel tube and computed its heat transfer correlations. The accuracy of the heat transfer correlation was verified using a parallel-tube heat transfer test bench with air as the working fluid. Liu et al. [6] proposed a mathematical model for a double-layer spiral-wound heat exchanger and experimentally verified the accuracy of the theoretical model. Neeraas et al. [7] conducted an experimental analysis of the falling-film characteristics of the shell side of a tubular heat exchanger, and compared the results with the calculation method for the heat transfer coefficient proposed by Bays and McAdams [8]. Ho et al. [9, 10] conducted an experimental study on the heat transfer characteristics of a coil-wound heat exchanger and proposed its applicability. On the premise of considering the effect of viscosity on the heat transfer coefficient of the shell side, Srbislav et al. [11] obtained a new empirical formula for calculating the heat transfer coefficient. Wu et al. [12, 13] analyzed tube-wound heat transfer. Wang and Jian et al. [14–16] studied the influence of geometric parameters on the heat transfer performance of a spiral-wound heat exchanger based on a multiobjective genetic algorithm and studied the influence of the long and short axes of an elliptical tube on the heat transfer performance using numerical simulation. Elliptical tube was found to have better heat transfer capacity than an ordinary circular tube. In [17], the single-phase heat transfer model of the shell side of an LNG spiral-wound heat exchanger was optimized by adding a viscosity correction term, and the influence of structural parameters on the heat transfer characteristics of the spiral-wound heat exchanger was analyzed. The results showed that the optimized model had a higher accuracy, and the heat transfer effect of the shell side of the spiral-wound heat exchanger could be significantly enhanced by reducing the thickness of the gasket and the radial spacing of the diameter. Yu et al. [18] studied the condensation heat transfer and frictional pressure drop characteristics in helically coiled tubes using experimental methods and compared their correlations. Yu et al. [19, 20] also studied the condensation flow patterns and heat transfer coefficients of methane/propane and ethane/propane mixtures in helical coils using numerical simulations and proposed a flow pattern division criterion for hydrocarbon refrigerant condensation. Qiu et al. [21, 22] combined computational fluid dynamics (CFD) with an improved silver method to calculate the condensation heat transfer coefficient of a mixed refrigerant in the tube of a spiral-wound heat exchanger. The simulation results indicated that the mass flow rate had a significant influence on the heat transfer coefficient of the mixed refrigerant.

When a spiral-wound heat exchanger is applied to an offshore LNG-FPSO, owing to the influence of sea conditions, the spiral-wound heat exchanger exhibits liquid bias, which deteriorates its heat transfer performance. Li et al. [23] found that the anti-sloshing ability of circular tubes was better than that of heat exchange tubes with elliptical cross-sections. By establishing a numerical model of flow boiling, Li et al. [24] found that sway conditions weakened heat transfer, and surge conditions promoted the separation of the liquid film and tube wall. Sun et al. [25] experimentally analyzed the influence of different sloshing conditions on the pressure drop and heat transfer performance of a heat exchanger. The fluctuation in the pressure drop was the largest under compound heaving and pitching conditions. Dong et al. [26] conducted experimental research on the rolling, pitching, yawing, and other sloshing conditions. Pitching was found to have the most significant influence on the heat transfer performance of the heat exchanger.

With technological innovations, some large-scale and high-cost scientific research difficulties need to be overcome. Because of the aforementioned problems, it is impossible to solve them through experimental research in real production. Numerical simulations are gradually being recognized by an increasing number of researchers owing to their low cost, short calculation cycle, and high accuracy. Currently, the research on spiral-wound heat exchangers is not mature. The problems of single-phase convective heat transfer, two-phase falling-film heat transfer, shell-side falling-film flow pattern change, and the influence of structural parameters on the spiral-wound heat exchanger have not been solved. The structure of a spiral-wound heat exchanger is quite different (the heat exchange tube is spirally arranged) from that of a conventional shell-and-tube heat exchanger, and thus it cannot be accurately described by the previous empirical correlation. Due to the high cost of new experimental manufacturing and the difficulty of measurement, experimental data on spiral-wound heat exchangers are lacking. This study uses a numerical simulation method to propose a suitable model by comparing it with experimental data. Based on land-based working and sloshing conditions, a sensitivity analysis of the structural parameters, such as the outer diameter of the winding pipe, winding angle, and pipe spacing, was performed.

## 2. Numerical approach

### 2.1 Geometric model and boundary conditions

**Fig 2:**
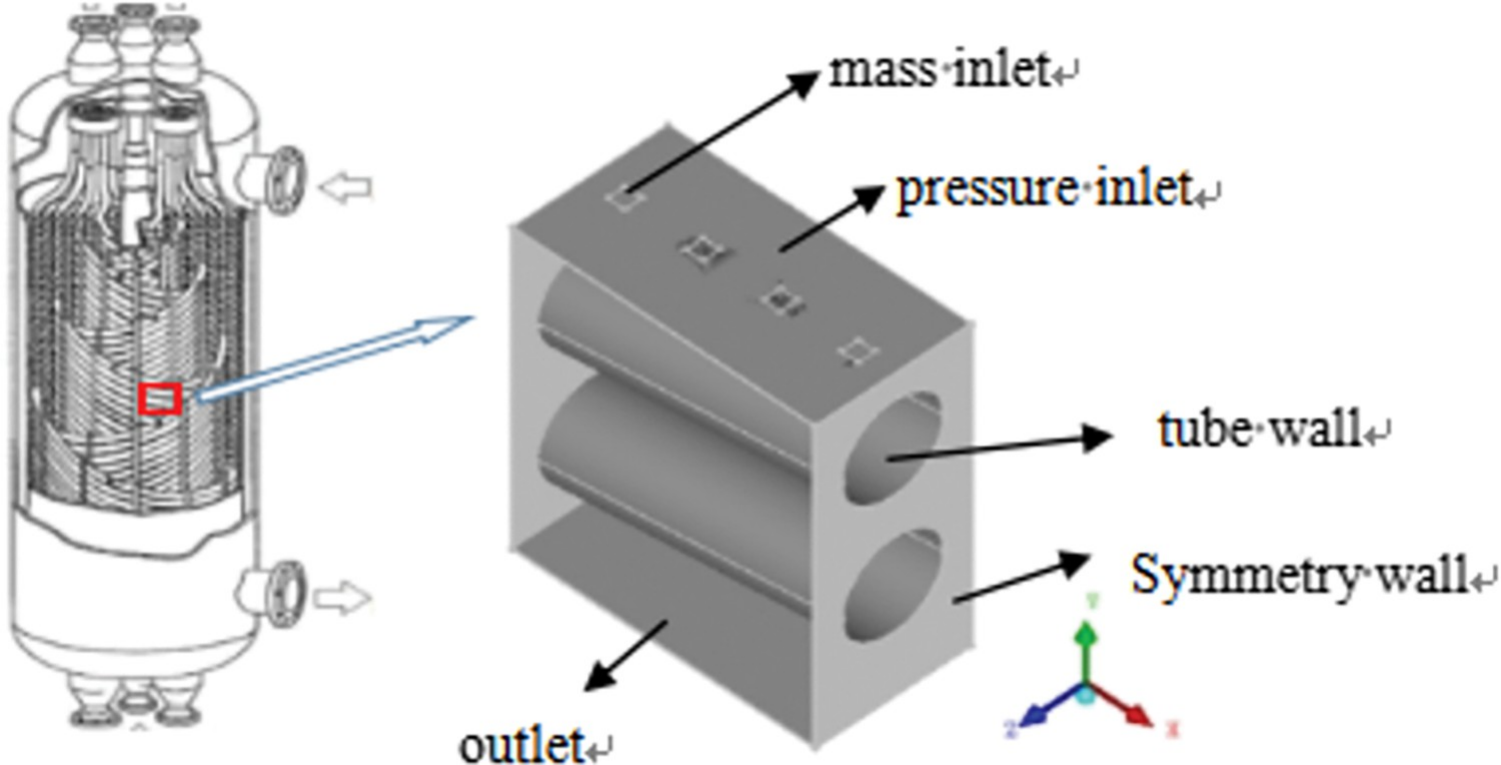
The model for three dimensional numerical simulation.

The physical model selected for this study is illustrated in Fig 2. Under the action of gravity, the shell-side refrigerant is sprayed from the nozzle onto the heat-exchange tube, and a liquid film is formed on the surface of the heat-exchange tube. The liquid film spreads along the axial and circumferential directions of the heat exchange tube, accumulates at the bottom of the tube, and flows downward.

In the numerical simulation, propane was used as the working fluid, and the VOF multiphase flow, evaporation condensation mass transfer, and CSF surface tension models were used for the simulation calculation. Gaseous propane was set to be the primary phase and liquid propane as the secondary phase. Initially, the entire computational domain was filled with gaseous propane, and the volume fraction of liquid propane at the inlet was 1. Considering the influence of contact angle on the film distribution, the contact angle was set to 10°.

### 2.2 Establishment of control equation

#### 2.2.1 Continuity equation

Gas phase:

$$\frac{\partial }{\partial t}(\alpha_{v}\rho_{v})+\nabla (\alpha_{v}\rho_{v}v_{v})=S_{m}$$ (1)

Liquid phase:

$$\frac{\partial }{\partial t}(\alpha_{l}\rho_{l})+\nabla \cdot (\alpha_{l}\rho_{l}v_{l})=-S_{m}$$ (2)

$$\alpha_{v}+\alpha_{l}=1$$ (3)

where *α*_*v*_ *and α*_*l*_ are the volume fractions of gas and liquid fluids, respectively; *ρ*_*v*_ and *ρ*_*l*_ are the density of gas and liquid fluids, kg∙m^-3^; *S*_*m*_ is the source phase of mass transfer mass, kg.

When the fluid temperature is higher than the saturation temperature, the fluid will change from liquid to gas and evaporate. When the fluid temperature is lower than the saturation temperature, the fluid will change from gas to liquid and condense.

$$T_{l}>T_{sat}\;{\dot{m}}_{l\to v}=coeff\cdot \alpha_{l}\rho_{l}\frac{\left(T_{l}-T_{sat}\right)}{T_{sat}}$$ (4)

$$T_{l}<T_{sat}\;{\dot{m}}_{v\to l}=coeff\cdot \alpha_{v}\rho_{v}\frac{\left(T_{v}-T_{sat}\right)}{T_{sat}}$$ (5)

$$coeff=\frac{6}{d_{b}}\beta \sqrt{\frac{M}{2\pi RT_{sat}}}h_{fg}\frac{\alpha_{v}\rho_{v}}{\rho_{l}-\rho_{v}}$$ (6)

$$S_{m}={\dot{m}}_{l\to v}-{\dot{m}}_{v\to l}$$ (7)

where *T*_*l*_ is temperature of liquid, K; *T*_*sat*_ is the saturation temperature, K; *m*_*l→v*_ and *m*_*v→l*_ are the gasification quality and condensation quality, kg. *coeff* is evaporation condensation coefficient; *d*_*b*_ is the bubble diameter, m; *β* is the adaptation coefficient of gas, *β* = 1; *M* is mass flow, kg∙s^-1^; *R* is is the radius of heat exchange tube, m; *h*_*fg*_ is the latent heat of vaporization, kJ·kg^-1^_._

#### 2.2.2 Momentum equation

$$\frac{\partial (\rho v)}{\partial t}+\nabla \cdot (\rho vv)=-\nabla p+\nabla [\mu (\nabla v+\nabla v^{T})-\frac{2}{3}\mu \cdot \nabla vI]+S_{F}$$ (8)

$$S_{F}=\rho g+F_{\sigma }$$ (9)

where *S*_*F*_ is the generalized body force; *F*_*σ*_ is the surface tension, N∙m.

#### 2.2.3 Energy equation

$$\frac{\partial (\rho E)}{\partial t}+\nabla \cdot [v(\rho E+p)]=\nabla \cdot (k\nabla T)+Q$$ (10)

$$Q=h_{fg}S_{m}$$ (11)

where *Q* is latent heat, kJ.

#### 2.2.4 Dynamic mesh mathematical model of sloshing boundary conditions

**Fig 3:**
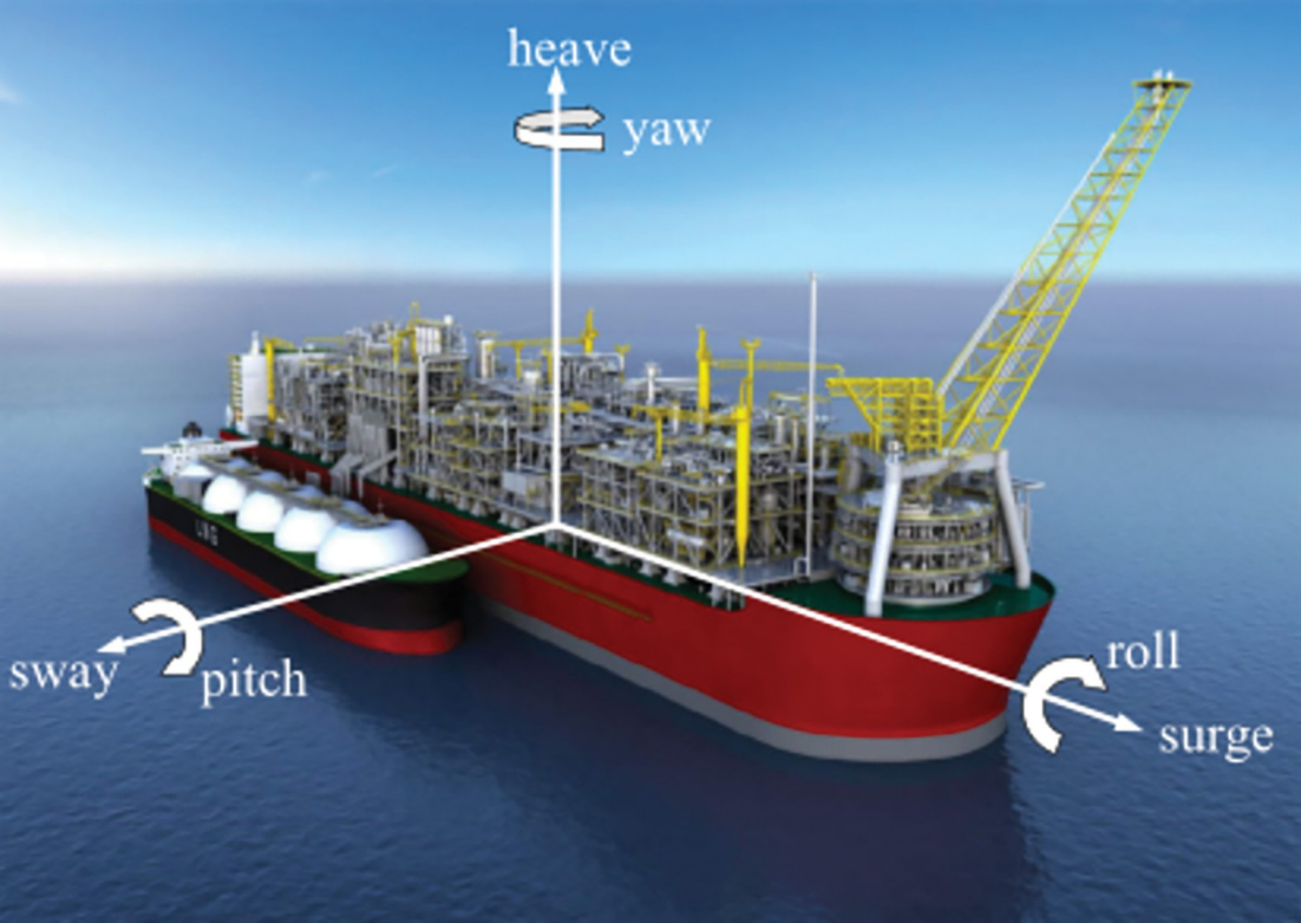
Movement of FLNG ship.

An FLNG ship floats at sea. It produces motion with six degrees of freedom and is affected by waves, wind, and other factors in the marine environment. As shown in Fig 3, surge (x-direction), sway (y-direction), and heave (z-direction) are linear motions. Roll (x-direction), pitch (y-direction), and yaw (z-direction) are angular motions. In actual ocean conditions, owing to the irregularity of wave motion, the motion of a ship is not a repetition of a single motion, but a combination of multiple forms of motion.

For complex sea conditions, the string equivalent wave method is usually used to simplify the sea conditions. Hoerner et al. [27] verified the applicability of the simplified method:

$$P_{0}=a_{w}\cdot \sin(\omega_{e}t+\varphi )$$ (12)

Where *P*_0_ is the location of the research object, *a*_*w*_ is fluctuation margin, *w*_*e*_ is frequency, *φ* is the angle.

The angular velocity of Eq (12) with respect to time is as follows:

$$\omega_{i}=\frac{\theta_{\max}\pi }{180}\cdot \frac{2\pi }{T}\cos(\frac{2\pi }{T}\cdot t)$$ (13)

Where *i* represents any direction of the x, y, and z axes, *θ*_*max*_ is sloshing amplitude, *T* sloshing period, *t* is the time value.

The linear velocity can be expressed as:

$$v_{i}=L\cdot \frac{2\pi }{T}\cos(\frac{2\pi }{T}\cdot t)$$ (14)

Where *L* is sloshing displacement.

### 2.3 Grid division and grid independence

**Fig 4:**
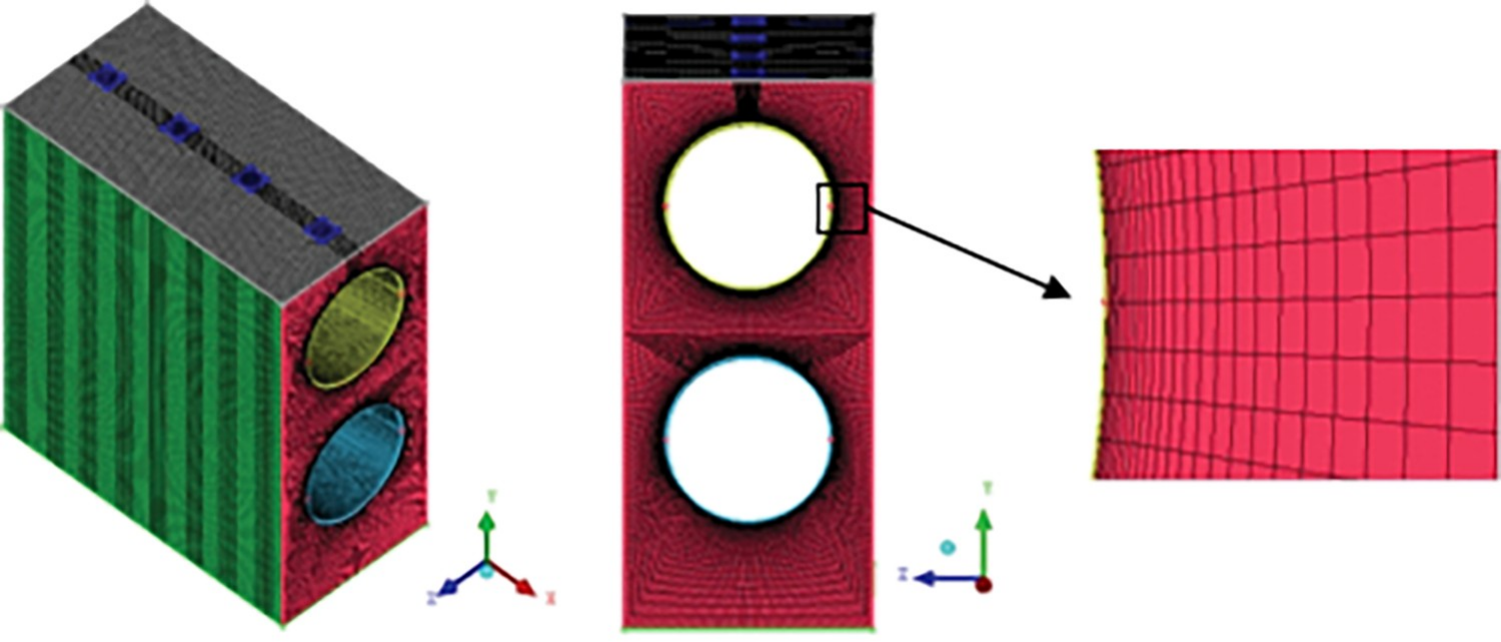
The grid model for three dimensional numerical simulation.

Using CFD software and structured grid drawing method, the calculation model was divided into grids; the grids are shown in Fig 4. To capture the falling-film flow phenomenon, the grid was encrypted around the liquid inlet and shell-side tube. In the first grid layer, near the surface of the heat exchange tube, the minimum size was set to 0.02 mm. At a position far from the surface of the heat-exchange tube, the maximum mesh size was set to 1 mm.

**Fig 5:**
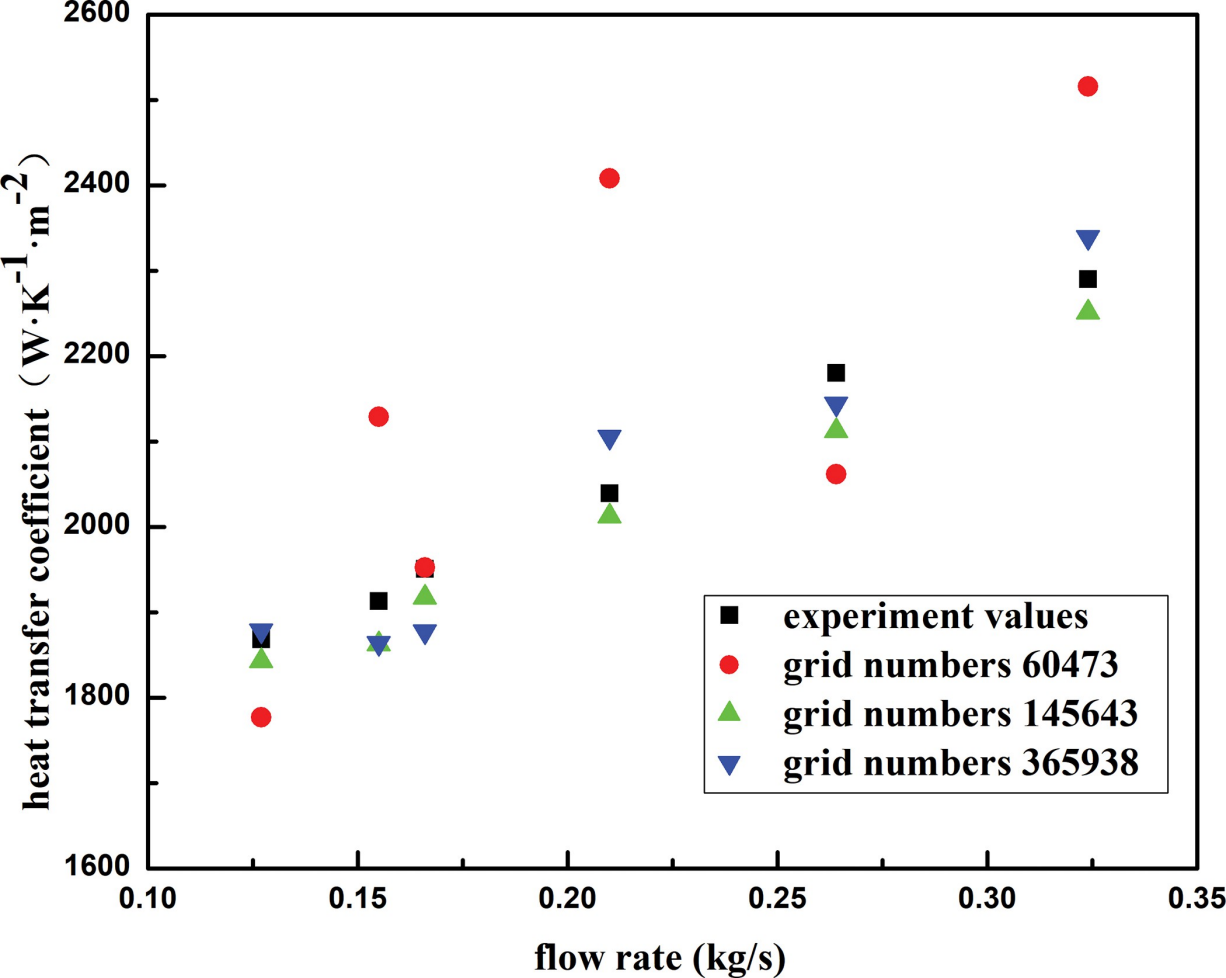
Test of independence.

Fig 5 shows the distribution of the simulated values of the heat transfer coefficient for different grid numbers and the experimental values. When the number of grids was in the range of 145,000–366,000, the heat transfer coefficient changed slightly, and the deviation was controlled within 0.5%. To accelerate the calculation speed, an actual numerical calculation was performed under the condition that the number of grids was 145,000; the results were less affected by the number of grids.

The numerical simulation results of this study were compared with the experimental data in Reference [28]. The experimental conditions are shown in Table 1.

**Table 1:** Experimental condition.

Pressure*10^−5^/Pa	Flow rate/kg·s^-1^	Gas phase	Heat flux/W·m^-2^	heat transfer coefficient/W·K^-1^·m^-2^
4.04	0.127	0	3950.99	1868.08
3.98	0.155	0	3950.99	1913.31
4.00	0.166	0	3950.99	1951.10
4.01	0.210	0	3950.99	2039.27
4.01	0.264	0	3950.99	2180.20
3.99	0.324	0	3950.99	2290.43

**Fig 6:**
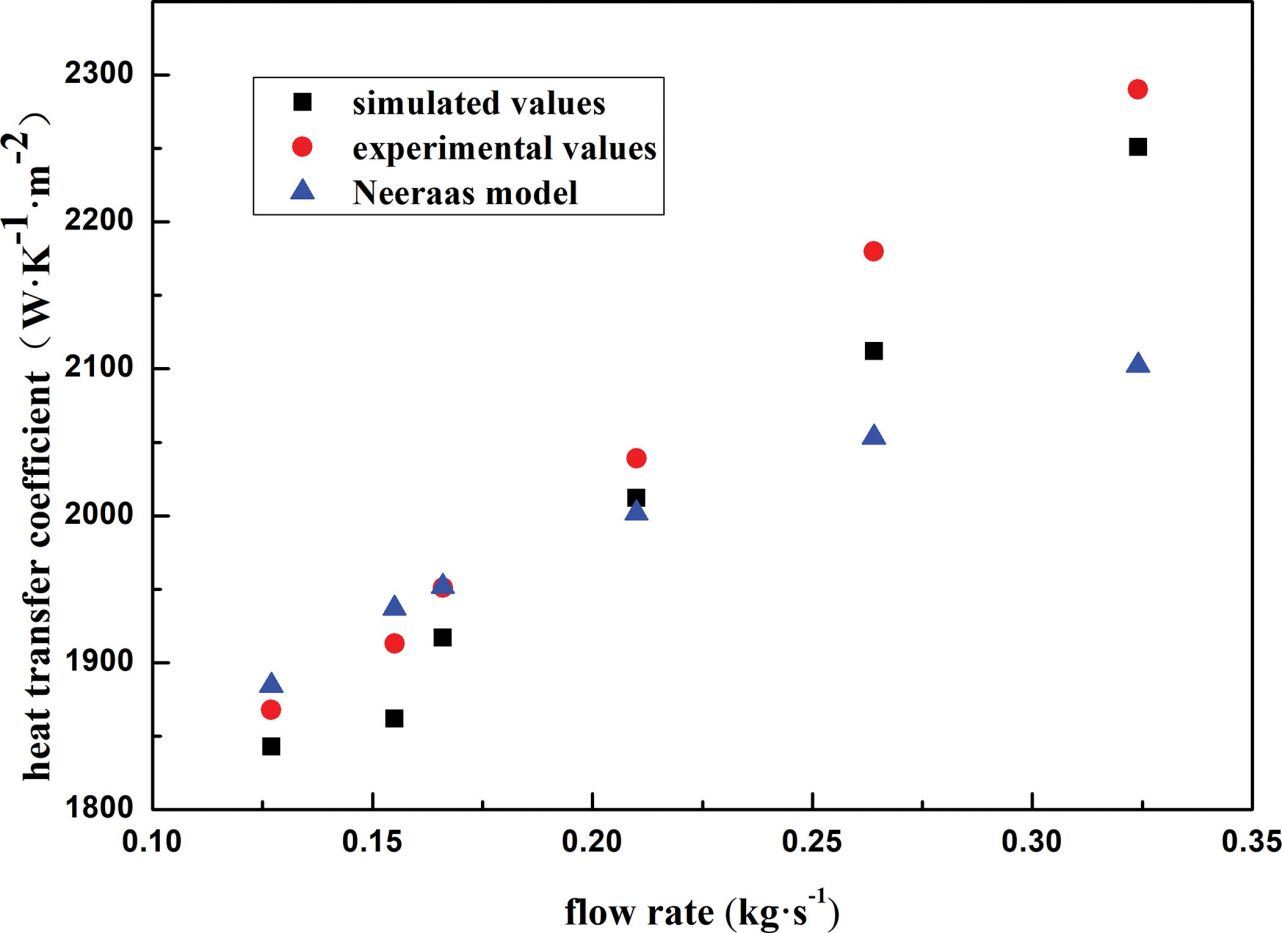
Heat transfer coefficient with different mass flow rate inlet.

Fig 6 shows a comparison between the numerical and experimental heat transfer coefficients measured under different inlet flow rates. The numerical and experimental values agree to within 6% and are in good agreement with the results obtained using the Neeraas shell-side two-phase heat transfer model. The numerical and experimental values differed by a maximum of 6.62% under a mass flow rate of 0.324 kg·s^-1^. Therefore, this model is suitable for simulation calculations.

**Fig 7:**
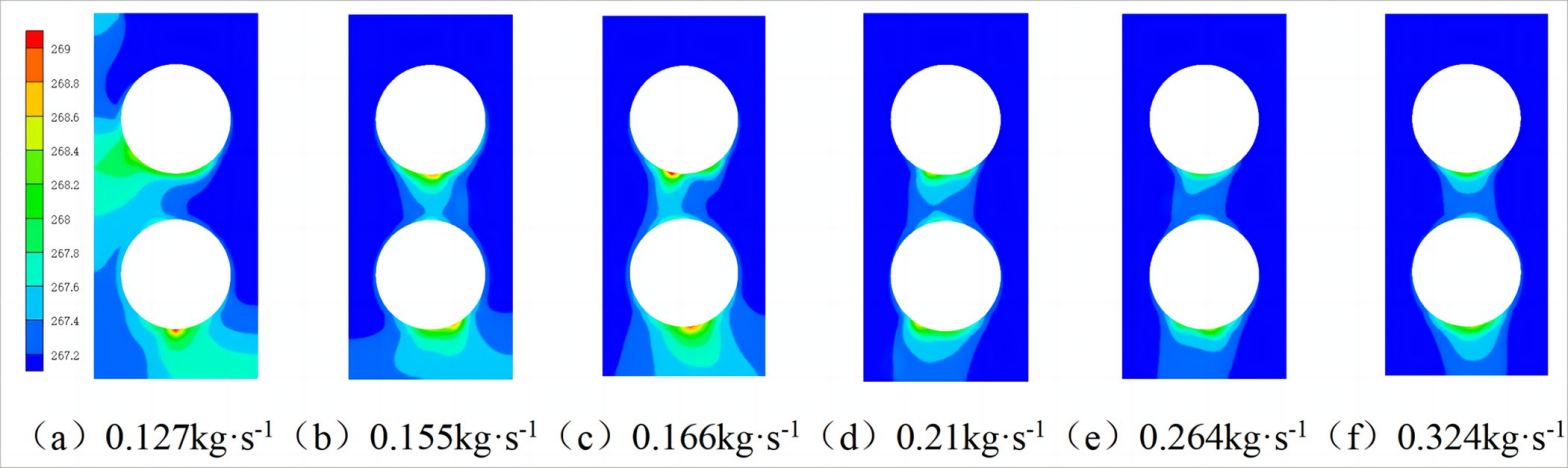
Radial temperature cloud diagram with different mass flow rate.

Fig 7 shows the radial temperature distribution of the spiral-wound heat exchanger under different mass flow rates. With an increase in the inlet shell-side refrigerant mass flow, the temperature difference between the inlet and outlet of the shell-side refrigerant decreases. This is because with an increase in the mass flow rate, the thickness of the liquid film formed by the shell-side refrigerant on the heat exchange tube gradually increases. However, under the action of gravity, the time for which the shell-side refrigerant remains in the heat-exchange tube remains essentially unchanged. Therefore, with an increase in the mass flow rate, the temperature difference between the inlet and outlet of the shell-side refrigerant gradually decreases.

**Fig 8:**
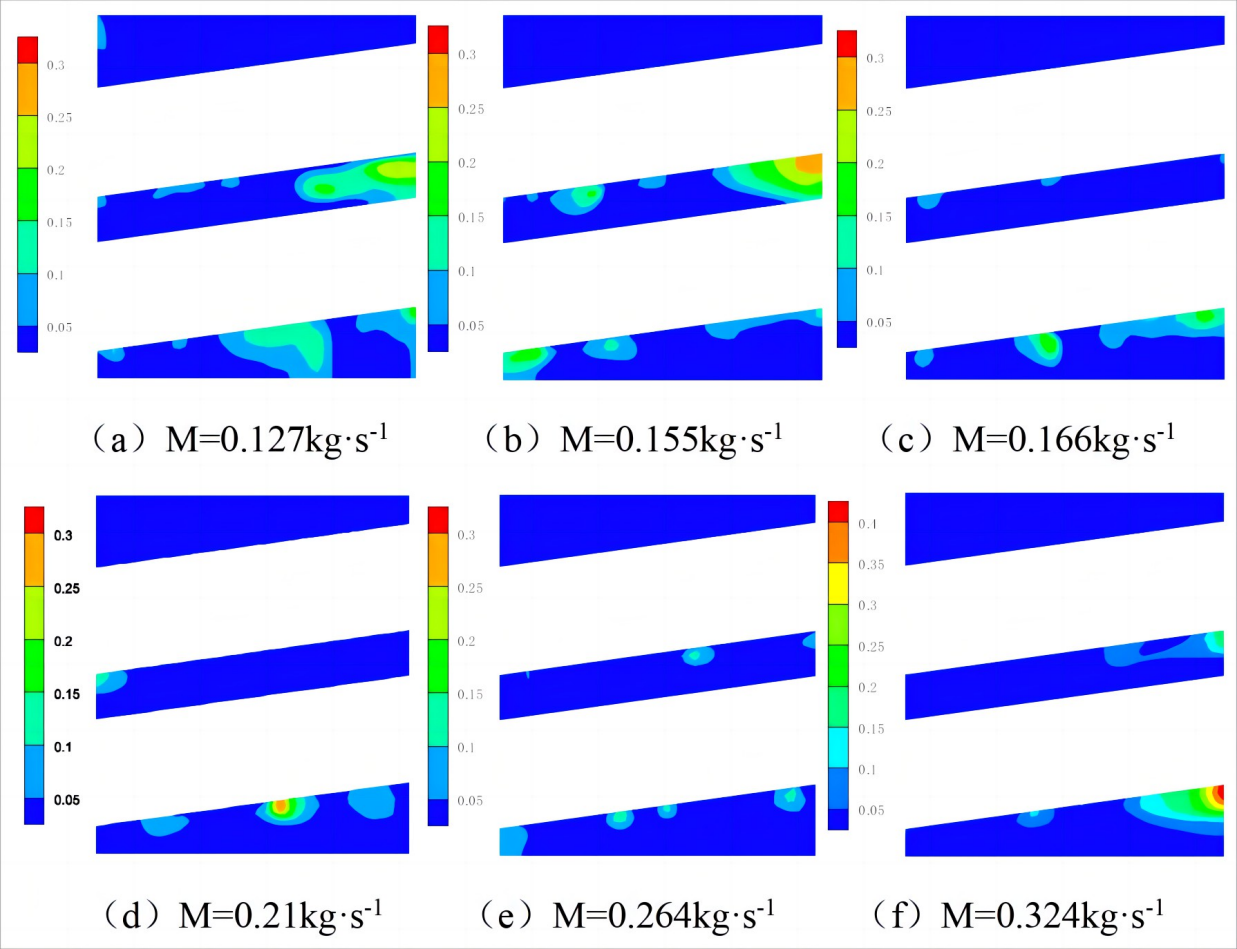
Radial gas phase volume fraction cloud diagram with different mass flow rate.

Fig 8 shows the axial gas fraction distribution of the heat exchanger under different mass flow rates. The proportion of gas-phase refrigerant decreases with an increase in the mass flow rate,. The proportion of gas-phase refrigerant on the right side of the heat exchanger is greater than that on the left side. This is because the heat flux of the heat exchange tube is constant, and the heat exchange between the heat exchange tube and shell-side refrigerant per unit time is certain. With an increase in the mass flow rate, the heat absorbed by the shell-side refrigerant is primarily used to increase the temperature of the shell-side refrigerant. Therefore, the proportion of gas-phase refrigerant decreases. Because the heat exchange tube is inclined, the liquid film formed by the shell-side refrigerant sprayed onto the heat exchange tube through the inlet shows a phenomenon of thick on the left side and thin on the right side. Therefore, the right-side refrigerant absorbs heat more easily to reach its gasification temperature and vaporize.

## 3. Analysis of simulation results of land-based conditions

The main structure of the shell side includes the inner diameter of the shell, outer diameter of the core, outer diameter of the winding tube, winding diameter, winding angle, axial winding-tube spacing, and radial winding-tube spacing. In this paper, winding angle *α*, outer diameter *d*, and axial tube spacing *P*_*l*_ are simulated and analyzed.

### 3.1 Influence of winding angle

To study the influence of winding angle on the heat transfer performance of spiral-wound heat exchanger, the heat transfer characteristics of a heat exchanger with winding angles of 5°, 8°, and 11° were simulated and analyzed.

**Fig 9:**
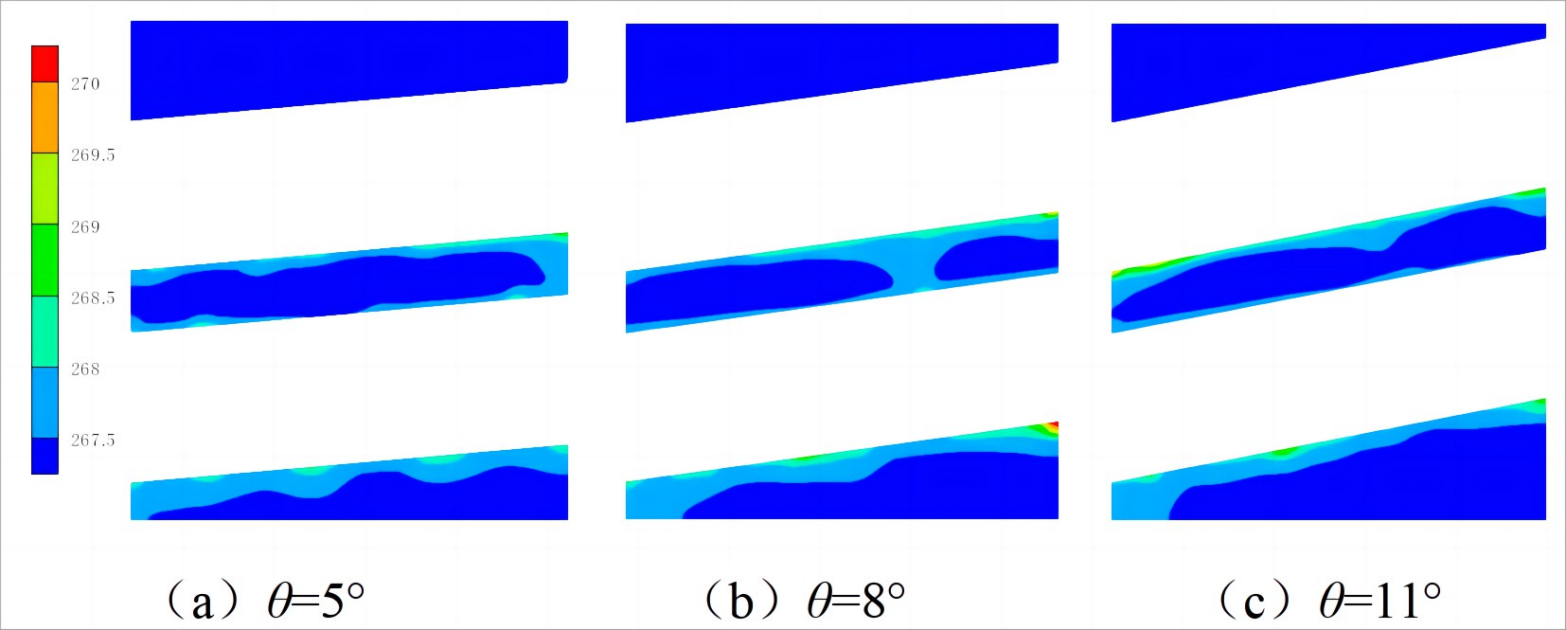
Axial temperature cloud diagram with different mass flow rate.

**Fig 10:**
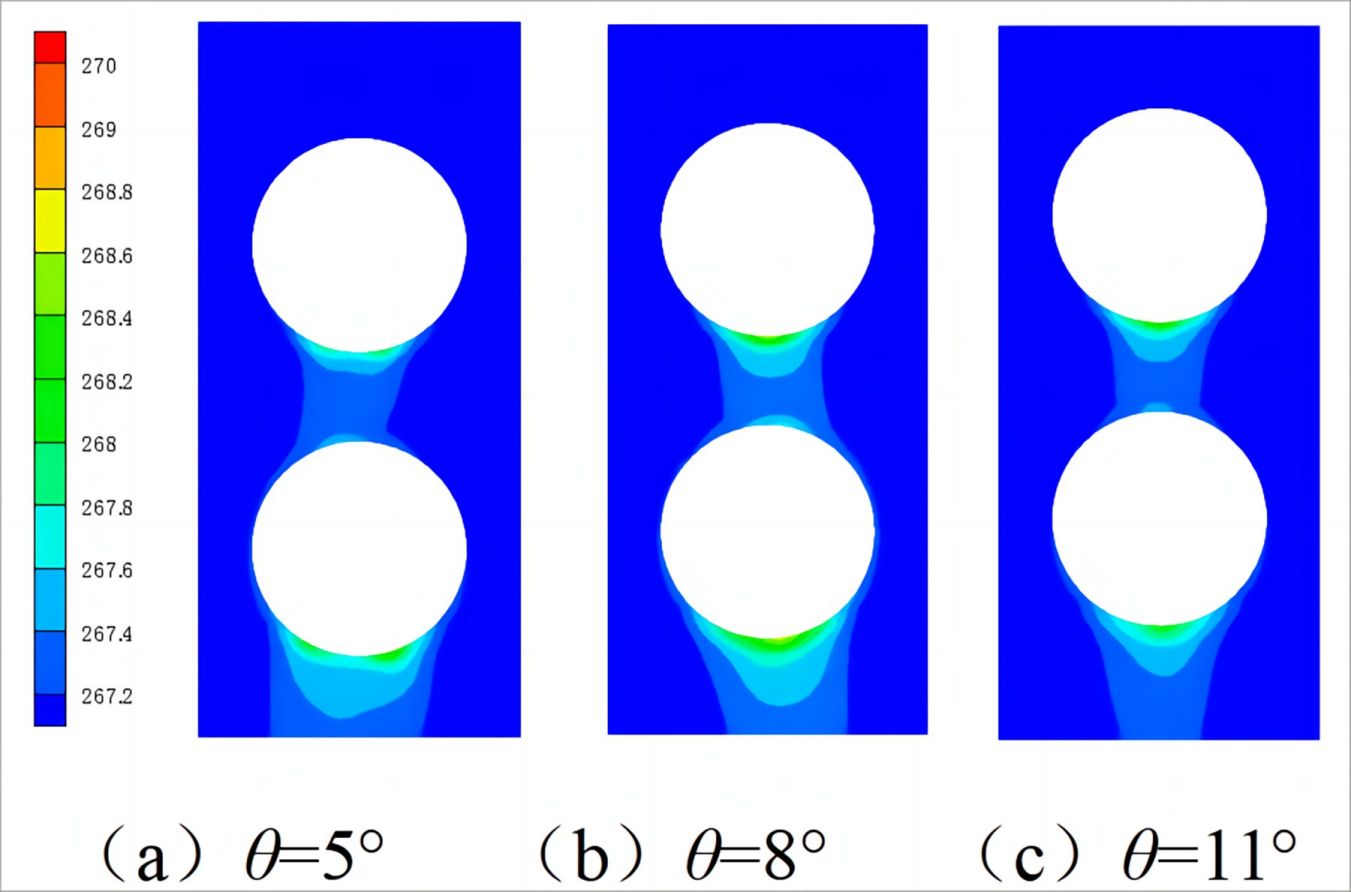
Radial temperature cloud diagram with different mass flow rate.

Figs 9 and 10 show the axial and radial temperature distributions of the spiral-wound heat exchanger under different winding angles. As shown in Fig 9, the right refrigerant temperature of the heat exchanger is higher, whereas the left refrigerant temperature is lower. This is because the heat exchange tube is inclined. Therefore, when the shell-side refrigerant is sprayed onto the heat exchange tube through the distributor, a liquid film is distributed on the heat exchange tube. Under the action of gravity, the low-temperature refrigerant flows to the left side of the heat exchange tube, resulting in the thickness of the liquid film on the left side being greater than that on the right side. As shown in Fig 10, as the winding angle increases, the inlet and outlet temperatures of the shell-side refrigerant decreases. This is because, as the winding angle increases, under the action of gravity, the residence time of the shell-side refrigerant on the heat exchange tube becomes shorter, and the low-temperature refrigerant can exchange heat more fully with the lower heat exchange tube.

**Fig 11:**
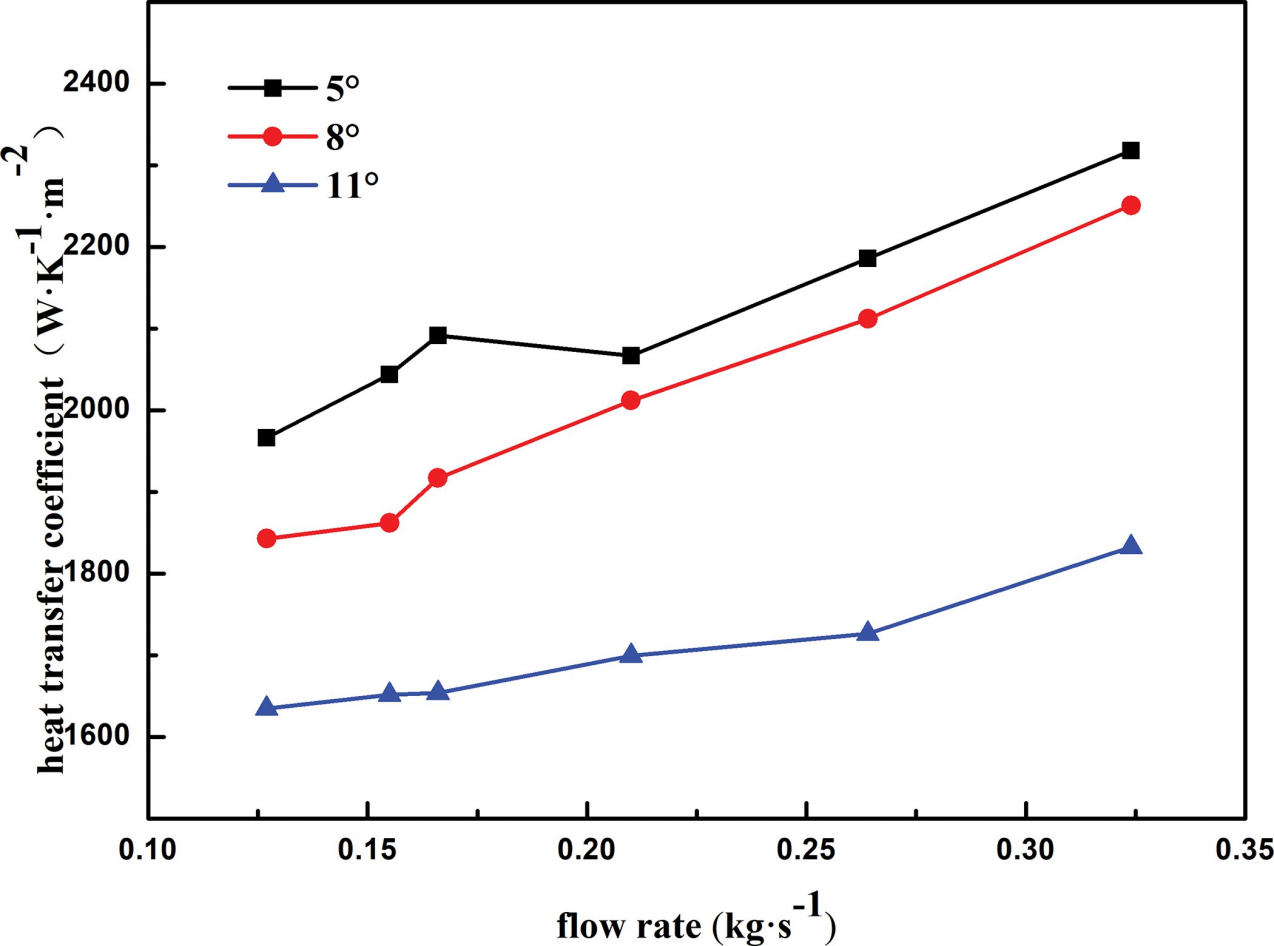
Heat transfer coefficient varies with mass flow rate of different winding angle.

Fig 11 shows the heat transfer coefficient of the shell side with respect to the mass flow rate for different winding angles. The variation trend of the heat transfer coefficient with the mass flow rate is generally the same for different winding angles. At the same mass flow rate, the heat transfer coefficient of the shell side decreases with an increase in the winding angle of the heat exchange tube. For instance, at a mass flow rate of 0.127 kg·s^-1^, when the winding angle increases from 5° to 8°, the heat transfer coefficient decreases by 6.70%. When the winding angle is constant, the heat transfer coefficient of the shell side increases with an increase in the mass flow rate. The mass flow rate of the shell-side refrigerant increases from 0.127 to 0.324 kg·s^-1^, and the mass flow rate increases by 2.5 times. At 5°, 8°, and 11°, the heat transfer coefficient of the shell side increases by 15.15%, 18.13%, and 10.79%, respectively. As the winding angle increases, the liquid film on the outer surface of the heat exchange tube accelerates the flow to the right side of the heat exchange tube under the action of gravity, resulting in a shorter contact time between the shell-side refrigerant and heat exchange tube. This results in insufficient heat transfer between the refrigerant and heat exchange tube; thus, the heat transfer coefficient decreases as the winding angle increases.

### 3.2 Influence of tube spacing

To study the influence of tube spacing on the heat transfer performance of spiral-wound heat exchanger, the heat transfer characteristics of a heat exchanger with tube spacings of 14, 17, and 20 mm were simulated and analyzed.

**Fig 12:**
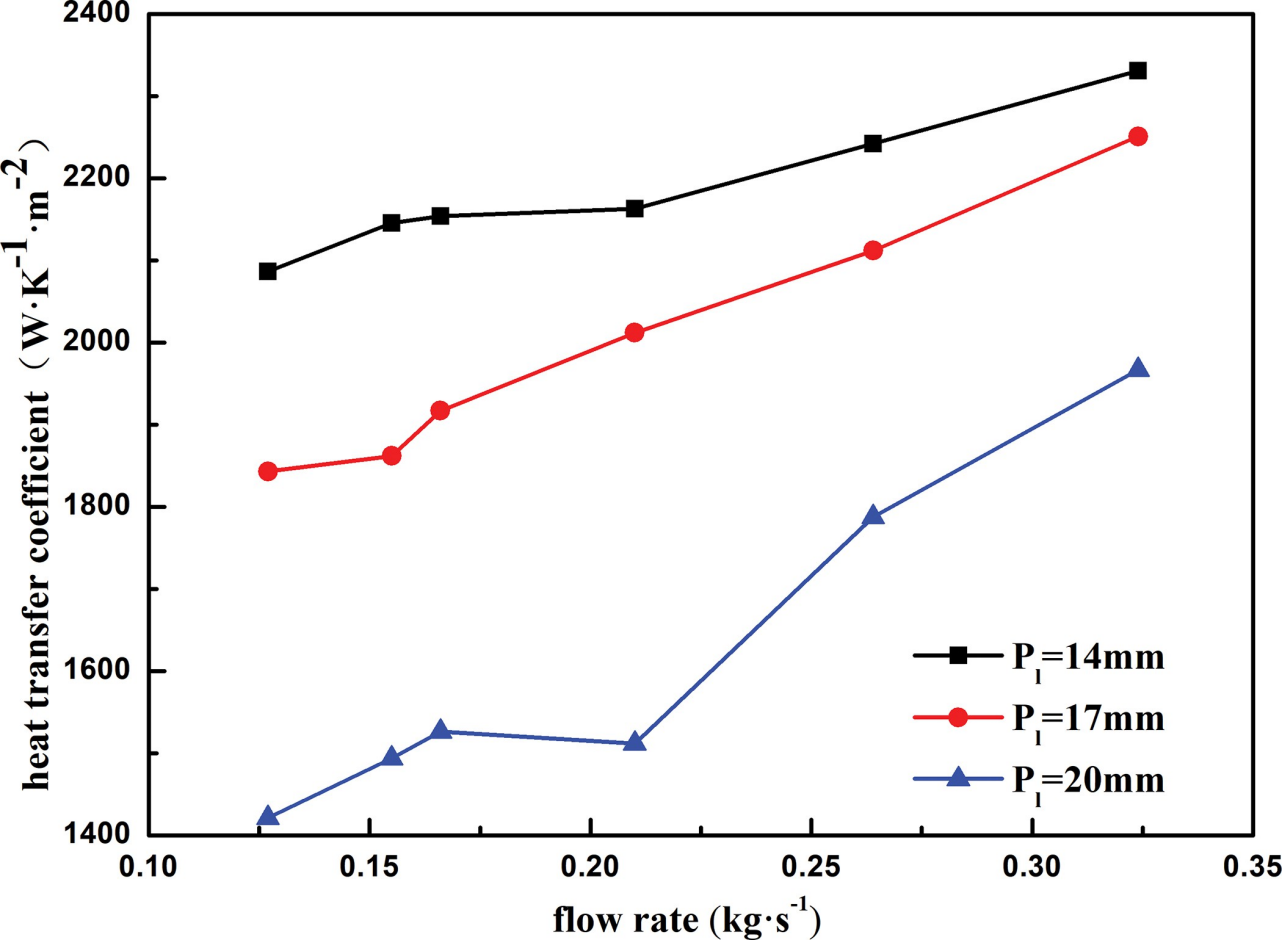
Heat transfer coefficient varies with mass flow rate of different longitudinal distance.

Fig 12 shows the heat transfer coefficient of the shell side with respect to the mass flow rate for different winding tube spacings. The variation trend of the heat transfer coefficient with the mass flow rate is generally the same for different tube spacings. Under the same mass flow rate, the heat transfer coefficient of the shell side decreases with an increase in the spacing of the heat exchange tube. At the same heat exchange tube spacing, the heat transfer coefficient increases with mass flow rate. As the heat exchange tube spacing increases from 14 to 17 mm, the shell side heat transfer coefficient decreases by 13.21% and the mass flow rate increases by 2.5 times. The heat transfer coefficient increases by 10.48%, 18.13%, and 27.73%, when the tube spacing is 14, 17, and 20 mm. As the distance between the heat exchange tubes increases, the velocity of the shell-side refrigerant from the first layer of the heat exchange tube to the second layer increases and the impact force of the shell-side refrigerant on the heat exchange tube becomes larger, resulting in the shell-side refrigerant not forming a stable liquid film on the outer surface of the heat exchange tube. Thus, the heat exchange between the shell-side refrigerant and heat exchange tube is insufficient, which weakens the heat exchange performance of the heat exchanger.

### 3.3 Influence of outer diameter of heat exchange tube

To study the influence of the outer diameter of the heat exchange tube on the heat transfer performance of a spiral-wound heat exchanger, we simulated and analyzed the heat transfer characteristics of a heat exchanger when the outer diameters of the heat exchange tube were 9, 12, and 15 mm.

**Fig 13:**
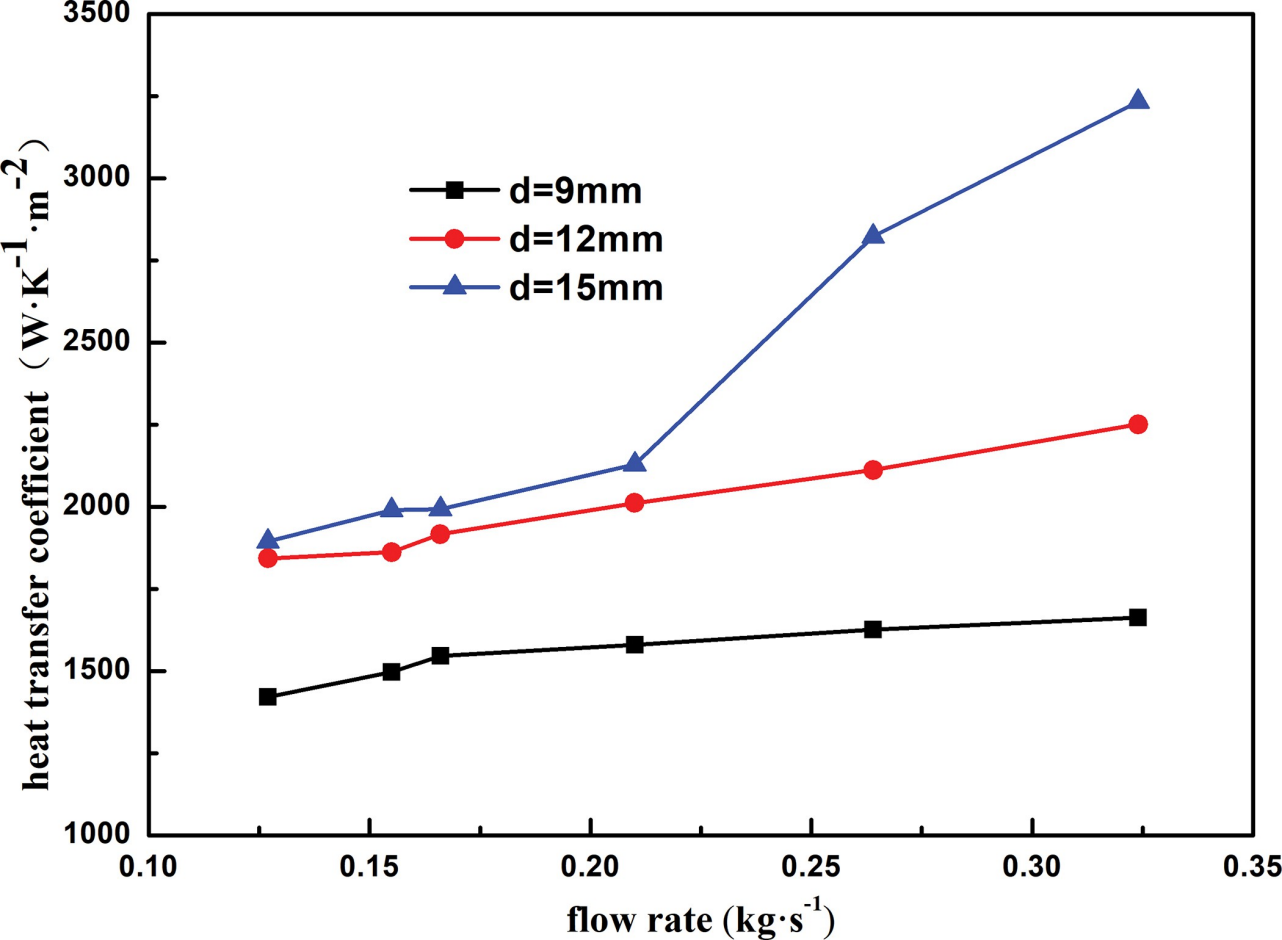
Heat transfer coefficient varies with mass flow rate of different outside tube diameter.

Fig 13 shows the heat transfer coefficient of the shell side with respect to the mass flow rate for different outer diameters of the heat exchange tubes. The variation trend of the shell-side heat transfer coefficient with the mass flow rate is generally the same for different outer diameters. At the same mass flow rate, the shell-side heat transfer coefficient decreases with an increase in the outer diameter of the heat-exchange tube. At the same outer diameter of the heat exchange tube, the heat transfer coefficient of the shell side increases with mass flow rate. As the outer diameter of the heat exchange tube increases from 9 to 12 mm, the heat transfer coefficient of the shell side increases by 22.89% And the mass flow rate increased by 2.5 times. The heat transfer coefficient increases by 14.55%, 18.13%, and 41.39% when the outer diameter of the heat exchange tube is 9, 12, and 15 mm, respectively. As the outer diameter of the heat exchange tube increases, the surface area of the contact between the shell-side refrigerant and heat exchange tube increases, which increases the heat exchange of the shell-side refrigerant and improves the heat exchange performance of the heat exchanger.

## 4. Effect of sloshing conditions on heat transfer of heat exchanger

In recent years, with the development and utilization of offshore natural gas, more attention has been paid to LNG-FPSO equipment. The influence of ship motion on natural gas liquefaction equipment needs to be addressed urgently. As a key component of natural gas liquefaction plants, it is important to study the flow and heat transfer characteristics of coiled tube heat exchanger under sloshing conditions. In this study, by writing UDF, dynamic mesh technology was used to simulate sloshing conditions in the computational domain. The gas-liquid two-phase flow model under sloshing conditions was solved to evaluate the influence of the sloshing conditions on the temperature field in the heat exchanger.

According to the literature [29, 30], the influence of the rolling and pitching motions on ships is more serious. The technology for reducing rolling has increasingly matured and improved; however, pitching has not been well solved. Therefore, this study investigated the influence of pitching on the heat transfer of a spiral-wound heat exchanger. 49 working conditions are simulated through the control variable method. The simulation conditions are listed in Table 2.

**Table 2:** Cases.

geometric parameter	Sloshing form	Sloshing amplitude *θ*/°	Sloshing period *T*/s
*α* = 5°、8°、11° *P*_*l*_ = 14mm、17mm、20mm *d* = 9mm、12mm、15mm	pitching	3	10
	pitching	5	10
	pitching	7	10
	pitching	9	10
	pitching	5	6
	pitching	5	15
	pitching	5	20

### 4.1 Effect of sloshing amplitude

In order to study the influence of sloshing angle on the shell-side heat transfer of spiral wound heat exchanger under different structural parameters, taking the sloshing period of 10 s and the sloshing amplitude of 3°, 5°, 7° and 9° as examples, the effects of different winding angles, heat exchange tube spacing and heat exchange tube outer diameter on the shell-side heat transfer of heat exchanger were numerically simulated by controlling variables.

#### 4.1.1 Winding angle

**Fig 14:**
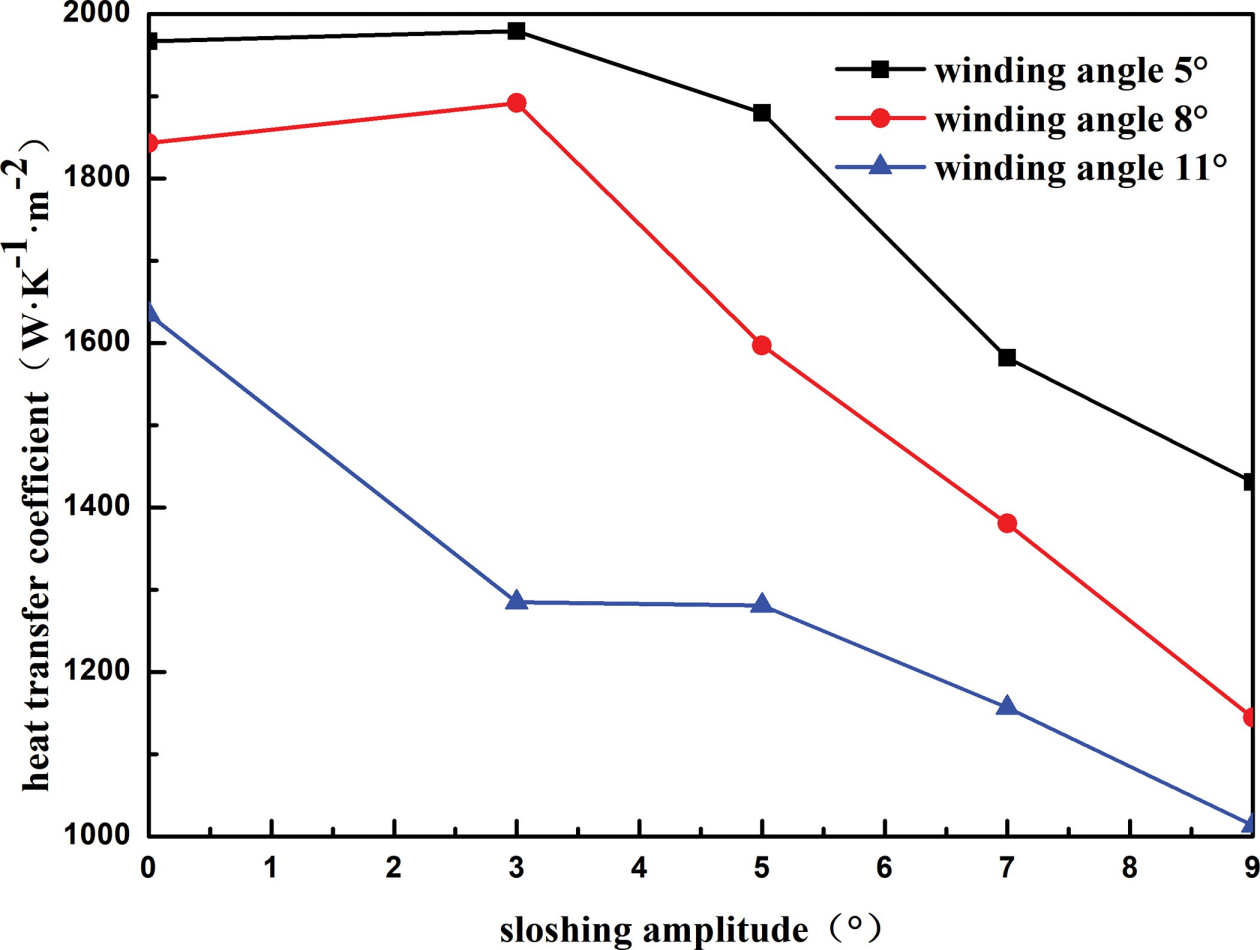
Heat transfer coefficient with sloshing amplitude of different winding angle.

Fig 14 shows the heat transfer coefficient of the heat exchanger shell side with respect to the sloshing angle for different winding angles. At a sloshing angle of 3°, the heat transfer coefficient increases when the winding angle is 5° and 8°, compared to the static condition. When the heat exchanger shakes at a small angle, the distribution of the shell-side refrigerant on the heat exchange tube is more reasonable, and the contact area between the shell-side refrigerant and the heat exchange tube increases, which enhances the heat transfer performance of the heat exchanger. At the same winding angle, the heat transfer coefficient of the shell side of the heat exchanger decreases with an increase in the sloshing angle. As the sloshing angle increases from 3° to 9°, the heat transfer coefficient of the shell side of the heat exchanger decreases by 27.69%, 29.84%, and 21.09% when the winding angle is 5°, 8°, 11°, respectively. At the same sloshing angle, the shell-side heat transfer coefficient decreases with an increase in the winding angle. For instance, at a sloshing angle of 3°, as the winding angle increases from 5° to 11°, the shell-side heat transfer coefficient decreases by 35.08%. This is because the larger the winding angle, the more the shell-side refrigerant is sprayed onto the outer surface of the heat exchange tube through the distributor. This makes it difficult to form a stable liquid film, which weakens the heat exchange between the refrigerant and heat exchange tube and reduces the heat transfer performance of the heat exchanger.

#### 4.1.2 Heat exchange tube spacing

**Fig 15:**
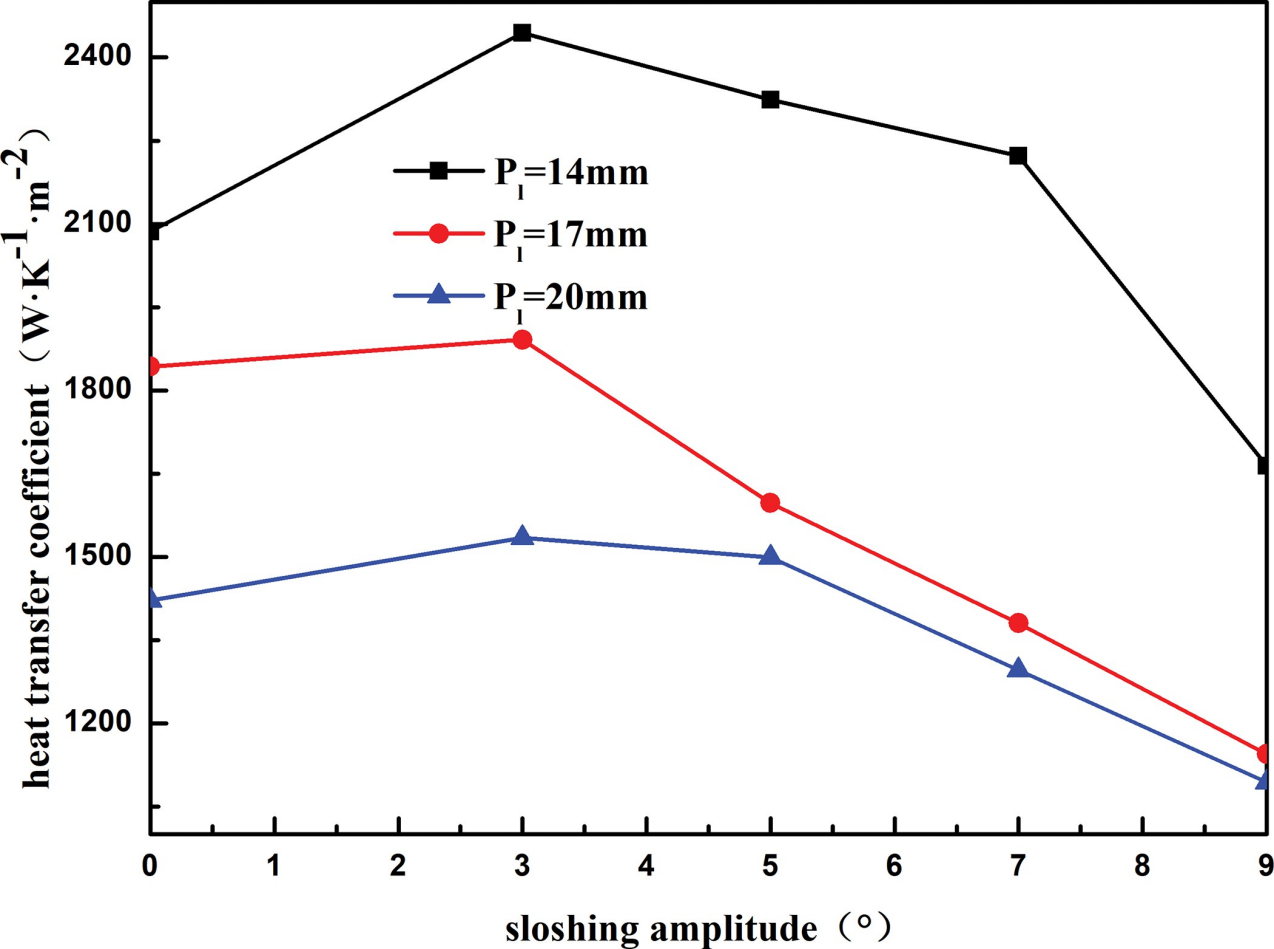
Heat transfer coefficient with sloshing amplitude of different longitudinal distance.

Fig 15 shows the heat transfer coefficient of the shell side of the heat exchanger with respect to the sloshing angle for different tube spacings. At the same heat exchange tube spacing, the heat transfer coefficient of the shell-side of the heat exchanger first increases and then decreases with an increase in the sloshing angle. The heat transfer coefficient of the shell side is the least when the sloshing angle is 7°. Sloshing weakens the heat transfer performance of the heat exchanger; when the sloshing angle increases from 3° to 9°, the shell-side heat transfer coefficient of the heat exchanger decreases by 43.48% at a heat exchange tube spacing of 14 mm. The heat transfer coefficient decreases by 29.84% and 28.71% when the heat exchange tube spacing is 17 mm and 20 mm, respectively. At the same sloshing angle, the heat transfer coefficient of the shell side decreases with an increase in the heat exchange tube spacing. For instance, at a sloshing angle of 3°, the heat transfer coefficient of the shell side decreases by 47.87% as the heat exchange tube spacing increases from 14 to 20 mm. This is because the shell-side refrigerant is sprayed onto the outer surface of the first layer of the heat exchange tube through the distributor and then drops to the outer surface of the second layer under the action of gravity. However, the larger the distance between the heat exchange tubes, the faster the shell-side refrigerant drops to the outer surface of the second layer, and the more difficult it is to form a stable liquid film on the outer surface of the heat exchange tube. This weakens the heat exchange between the refrigerant and heat exchange tube and reduces the heat transfer performance of the heat exchanger.

#### 4.1.3 Outer diameter of heat exchange tube

**Fig 16:**
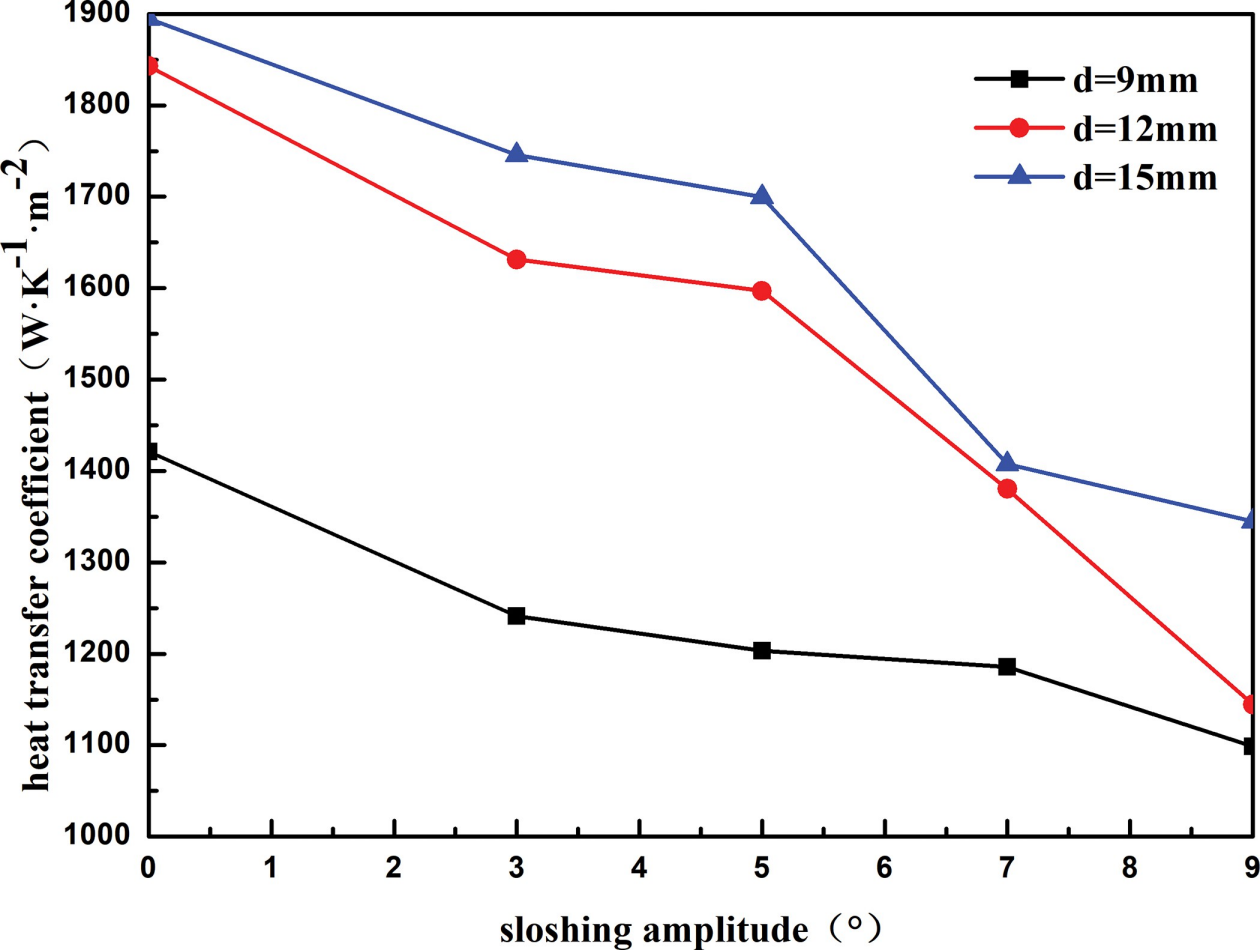
Heat transfer coefficient with sloshing amplitude of different outside tube diameter.

Fig 16 shows the heat transfer coefficient of the heat exchanger shell side with respect to the sloshing angle for different outer diameters of the heat exchange tubes. At the same outer diameter, the heat transfer coefficient of the shell side of the heat exchanger decreases with an increase in the sloshing angle. As the sloshing angle increases from 3° to 9°, the heat transfer coefficient of the shell side of the heat exchanger decreases by 11.47% at an outer diameter of the heat exchange tube of 9 mm. The heat transfer coefficient decreases by 29.84% and 22.98% when the outer diameter of the heat exchange tube is 12 and 15 mm, respectively. At the same sloshing angle, the heat transfer coefficient of the shell side increases with an increase in the outer diameter of the heat exchange tube. For instance, at a sloshing angle of 3°, as the outer diameter of the heat exchange tube increases from 9 to 15mm, the heat transfer coefficient of the shell side increases by 28.91%. This is because the shell-side refrigerant is sprayed onto the outer surface of the heat exchange tube through the distributor and is wrapped on the outer surface of the heat exchange tube in a liquid film. As the diameter of the heat exchange tube increases, the contact area between the shell-side refrigerant and heat exchange tube increases continuously, which enhances the energy exchange between the refrigerant and heat exchange tube and improves the heat transfer performance of the heat exchanger.

### 4.2 Effect of sloshing period

Based on the analysis of the influence of the sloshing angle on the heat transfer performance of the heat exchanger, this study further investigates the influence of the sloshing period on the heat transfer performance of the heat exchanger. Sloshing periods of 6, 10, 15, and 20 s were considered, with a sloshing angle of 5°. The effects of different winding angles, heat exchange tube spacing, and heat exchange tube outer diameter on the heat transfer of the shell side of the heat exchanger were numerically simulated by controlling variables.

#### 4.2.1 Winding angle

**Fig 17:**
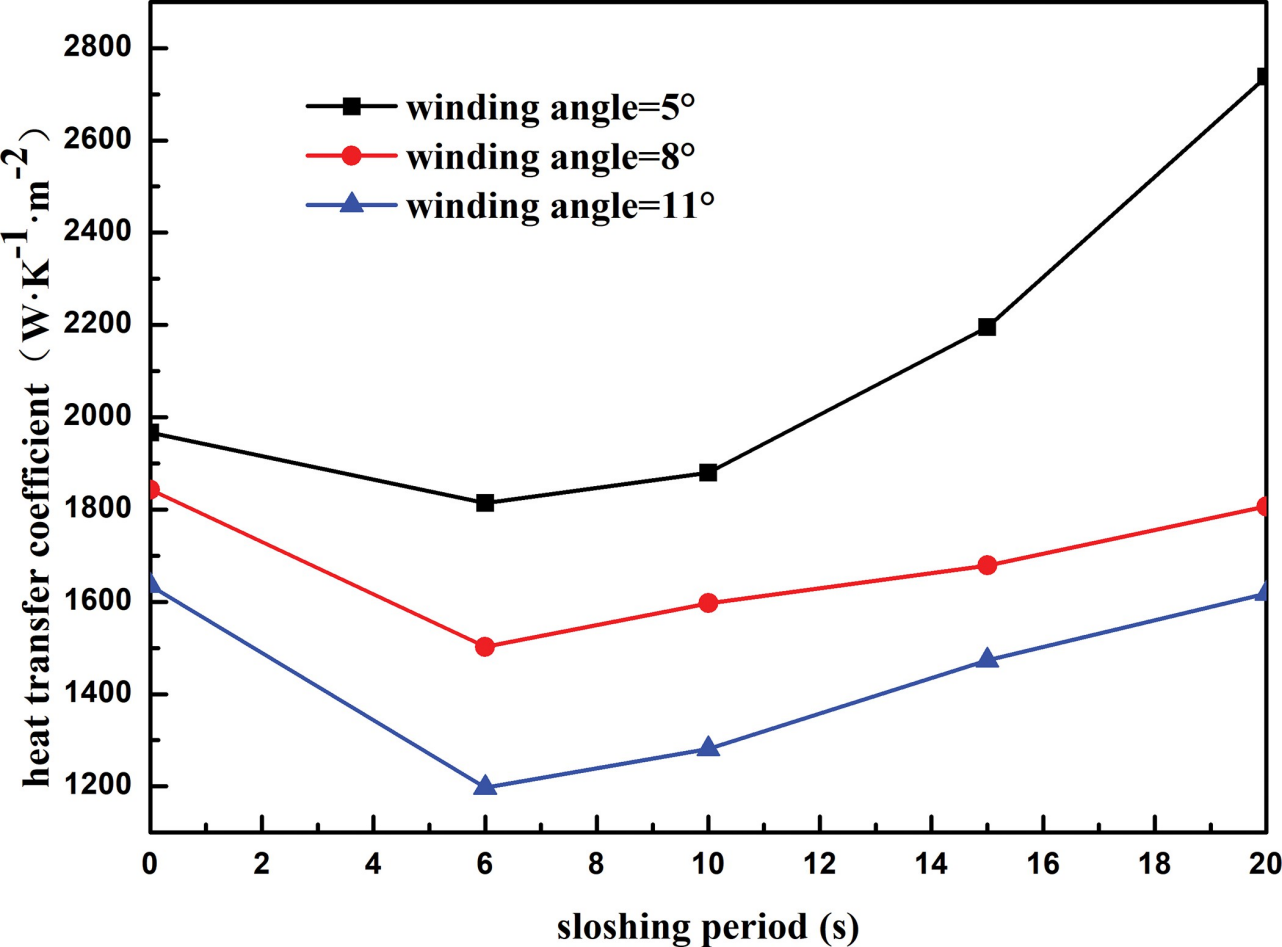
Heat transfer coefficient with sloshing period of different winding angle.

Fig 17 shows the heat transfer coefficient of the heat exchanger shell side with respect to the sloshing period for different winding angles. At the same winding angle, the heat transfer coefficient of the heat exchanger shell side first decreases and then increases with an increase in the sloshing angle. When the sloshing period exceeds 15 s, the effect of sloshing on the heat transfer of the heat exchanger shell side weakens. At a winding angle of 5°, as the sloshing period increases from 6 to 20 s, the shell-side heat transfer coefficient of the heat exchanger decreases by 33.75%. The shell-side heat transfer decreases by 16.86%, 26.01% at winding angles of 8° and 11°, respectively. At the same sloshing period, the shell-side heat transfer coefficient decreases with an increase in the winding angle. For instance, at a sloshing period of 6 s, as the winding angle increases from 5° to 11°, the shell-side heat transfer coefficient decreases by 34.00%. This is because with a larger winding angle, the shell-side refrigerant is sprayed onto the outer surface of the heat exchange tube through the distributor, making it difficult to form a stable liquid film. This weakens the heat exchange between the refrigerant and the heat exchange tube and reduces the heat transfer performance of the heat exchanger.

#### 4.2.2 Heat exchange tube spacing

**Fig 18:**
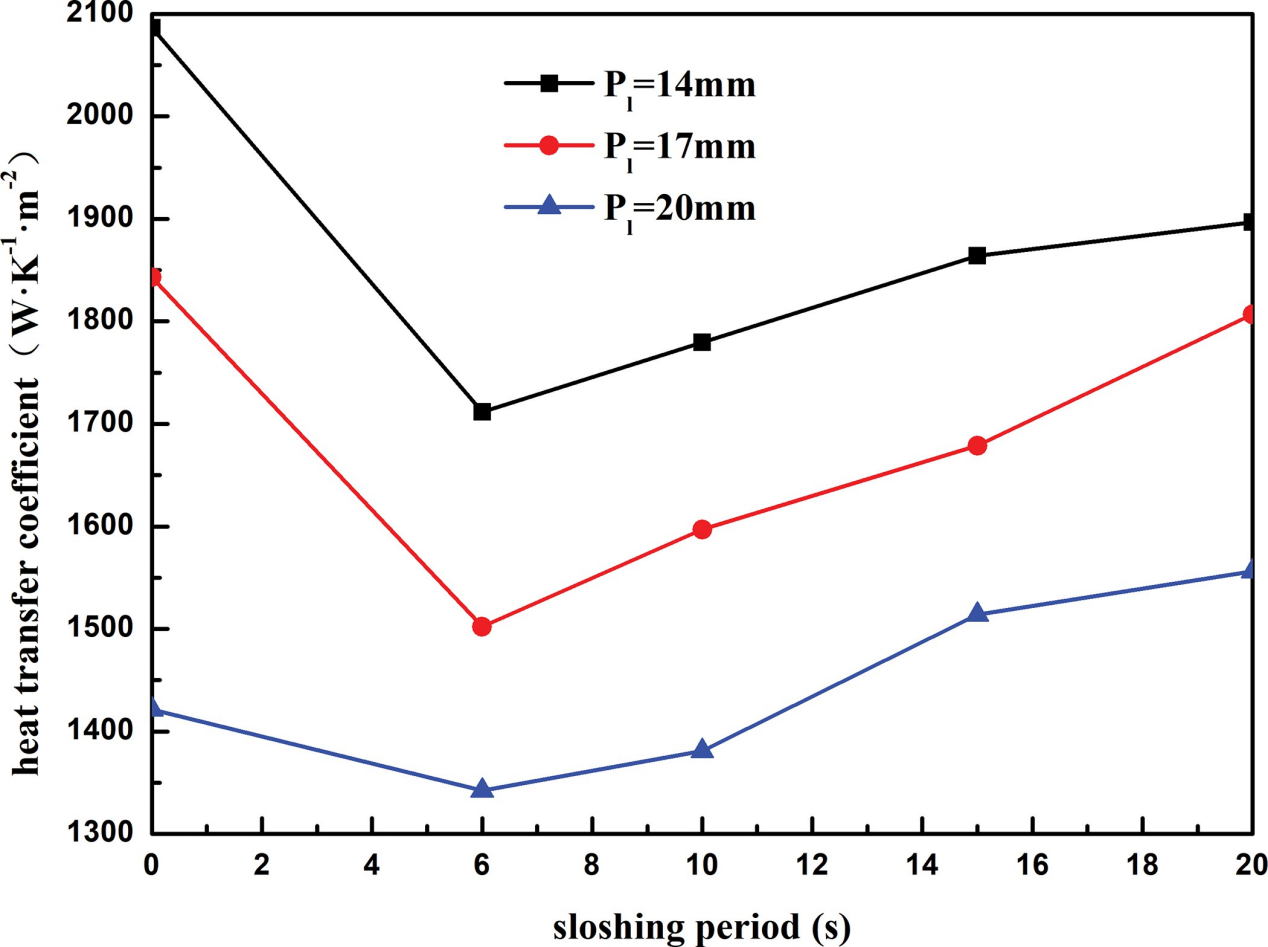
Heat transfer coefficient with sloshing period of different longitudinal distance.

Fig 18 shows the variation curve of heat transfer coefficient of shell side of heat exchanger with sloshing period under different tube spacing. It can be seen that when the heat exchange tube spacing is constant, the heat transfer coefficient of the shell side of the heat exchanger decreases first and then increases with the increase of the sloshing period. When the sloshing period is greater than 15 s, the effect of sloshing on the heat transfer of the shell side of the heat exchanger is relatively weak. When the sloshing period increases from 6s to 20s, the shell-side heat transfer coefficient of the heat exchanger with the heat exchange tube spacing of 14mm is reduced by 9.76%, the shell-side heat transfer coefficient of the heat exchanger with the heat exchange tube spacing of 17mm is reduced by 29.84%, and the shell-side heat transfer coefficient of the heat exchanger with the heat exchange tube spacing of 20mm is reduced by 13.75%. When the sloshing period is constant, the heat transfer coefficient of the shell side decreases with the increase of the heat exchange tube spacing. Taking the sloshing period of 6s as an example, the heat transfer coefficient of the shell side decreases by 21.57% when the heat exchange tube spacing increases from 14 mm to 20 mm. This is because the shell-side refrigerant is sprayed onto the outer surface of the first layer of heat exchange tube through the distributor, and then drops to the outer surface of the second layer of heat exchange tube under the action of gravity. However, the larger the distance between the heat exchange tubes, the faster the shell-side refrigerant drops to the outer surface of the second layer of heat exchange tube, and the more difficult it is to form a stable liquid film on the outer surface of the heat exchange tube, which weakens the heat exchange between the refrigerant and the heat exchange tube and reduces the heat transfer performance of the heat exchanger.

#### 4.2.3 Outer diameter of heat exchange tube

**Fig 19:**
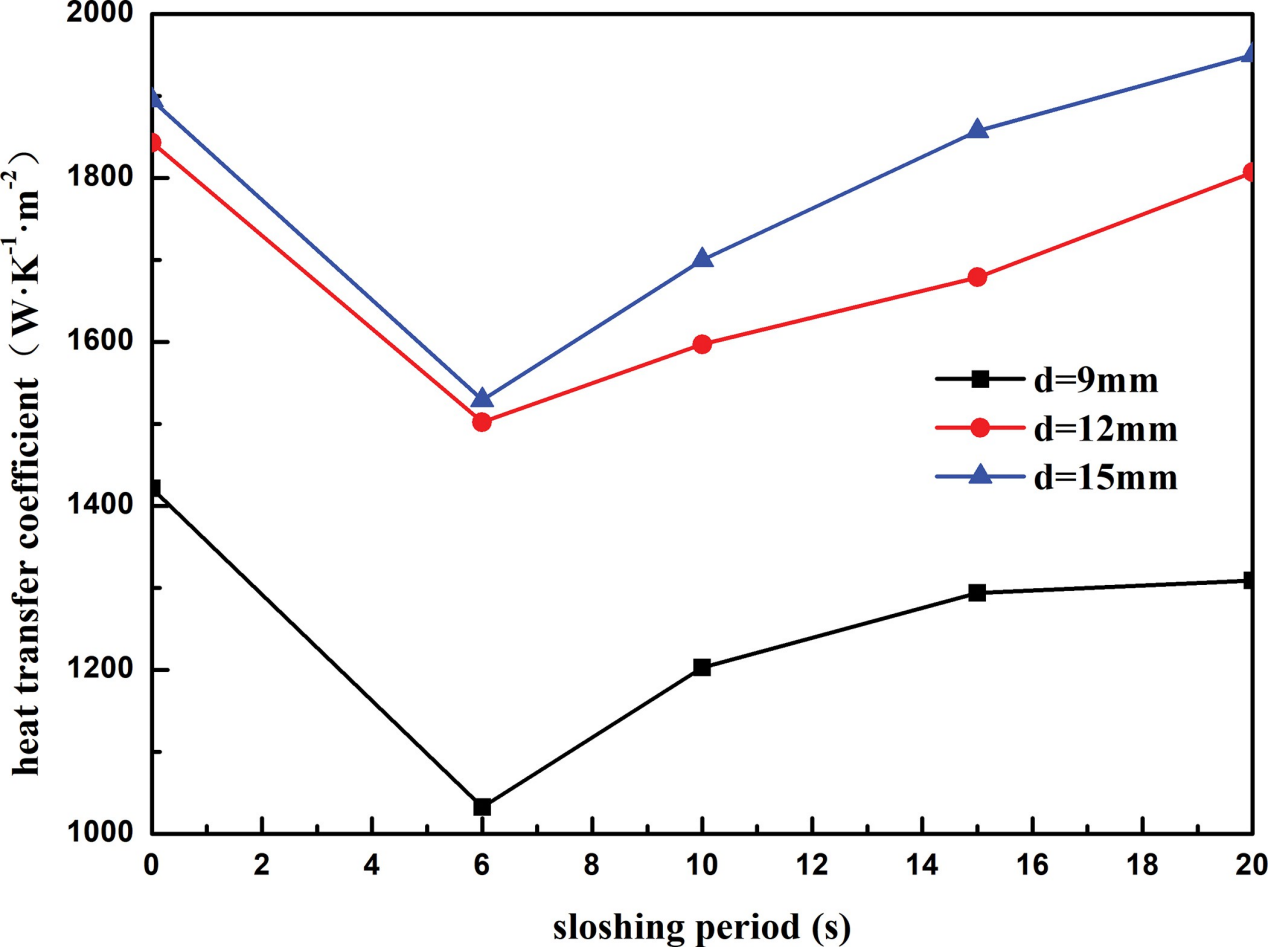
Heat transfer coefficient with sloshing period of different outside tube diameter.

Fig 19 shows the variation curve of heat transfer coefficient of heat exchanger shell side with sloshing period under different outer diameters of heat exchange tubes. It can be seen that when the outer diameter of the heat exchange tube is constant, the heat transfer coefficient of the heat exchanger shell side decreases first and then increases with the increase of the sloshing period. When the sloshing period is greater than 15 s, the effect of sloshing on the heat transfer of the heat exchanger shell side is relatively weak. When the sloshing period increases from 6s to 20s, the shell-side heat transfer coefficient of the heat exchanger with the outer diameter of the heat exchange tube of 9mm is reduced by 21.12%, the shell-side heat transfer coefficient of the heat exchanger with the outer diameter of the heat exchange tube of 12mm is reduced by 29.84%, and the shell-side heat transfer coefficient of the heat exchanger with the outer diameter of the heat exchange tube of 15mm is reduced by 21.57%. When the sloshing period is constant, the heat transfer coefficient of the shell side increases with the increase of the outer diameter of the heat exchange tube. Taking the sloshing period of 6s as an example, the outer diameter of the heat exchange tube increases from 9mm to 15mm, and the heat transfer coefficient of the shell side increases by 32.49%. This is because the shell-side refrigerant is sprayed onto the outer surface of the heat exchange tube through the distributor and is wrapped on the outer surface of the heat exchange tube in a liquid film. With the increase of the diameter of the heat exchange tube, the contact area between the shell-side refrigerant and the heat exchange tube increases continuously, which enhances the energy exchange between the refrigerant and the heat exchange tube and improves the heat transfer performance of the heat exchanger.

## 5. Conclusion

In this study, a three-dimensional mathematical model of a spiral-wound heat exchanger was established and compared with the experimental data in Reference [28]. The simulation results were in good agreement with the experimental results. Shell-side structural parameters affecting the heat transfer of the spiral-wound heat exchanger were simulated and analyzed. Based on land-based simulation conditions, a dynamic mesh mathematical model of sloshing boundary conditions was established. The effects of different structural parameters, such as the winding angle, heat exchange tube spacing, and outer diameter of the heat exchange tube, on the heat transfer performance of spiral-wound heat exchanger were analyzed under different sloshing amplitudes and periods. The main conclusions are as follows:

1. Under the land-based simulation conditions, the heat transfer coefficient of the shell side of the spiral-wound heat exchanger decreased with an increase in the winding angle of the heat exchange tube. As the winding angle increased from 5° to 8°, the heat transfer coefficient is reduced by 6.70%.;
2. Under the land-based simulation conditions, the shell-side heat transfer coefficient of the spiral-wound heat exchanger decreased with an increase in the heat exchange tube spacing. As the heat exchange tube spacing increased from 14 to 17 mm, the shell-side heat transfer coefficient decreased by 13.21%.
3. Under the land-based simulation conditions, the heat transfer coefficient of the shell side of the spiral-wound heat exchanger increased with an increase in the outer diameter of the heat exchange tube. As the outer diameter of the heat exchange tube increased from 9 to 12 mm, the heat transfer coefficient of the shell side increased by 22.89%.
4. For a fixed sloshing amplitude, the heat transfer coefficient of the wound heat exchanger decreased with an increase in the winding angle and spacing of the heat exchange tubes, and increased with an increase in the outer diameter of the heat exchange tubes. For fixed structural parameters of the heat exchanger, the heat transfer coefficient decreased with an increase in the sloshing amplitude. When the sloshing amplitude was less than 3°, sloshing promoted the heat transfer on the shell side. When the sloshing angle was above 7°, the heat transfer effect of the shell side deteriorated considerably, which weakened the heat transfer performance of the heat exchanger.
5. For a fixed sloshing period, the heat transfer coefficient of the wound heat exchanger decreased with an increase in the winding angle and spacing of the heat exchange tubes, and increased with an increase in the outer diameter of the heat exchange tubes. For fixed structural parameters of the heat exchanger, the heat transfer coefficient increased with sloshing period. When the sloshing period was longer than 15 s, the influence of sloshing on the heat transfer of the shell side of the heat exchanger was relatively weak.

This study investigated the heat transfer performance of a spiral-wound heat exchanger. Many aspects remain to be investigated, such as the flow characteristics of a spiral-wound heat exchanger (including the distribution of the liquid film outside the heat exchange tube along the tube wall, and the factors affecting the rupture of the liquid film), the flow pattern between heat exchange tubes and the basis of flow pattern conversion, and the influence of tube type, tube arrangement, and structural factors on the shell-side refrigerant flow.

## Supporting information

S1 File (DOCX)
