# Supplementary material for: Numerical simulation of heat transfer performance of spiral wound heat exchanger under sloshing condition
Source: PLoS One. 2023 Dec 11;18(12):e0295315. doi: 10.1371/journal.pone.0295315 (PMC10712881; doi:10.1371/journal.pone.0295315)
Supplement: S1 File — (DOCX) [file pone.0295315.s001.docx]

1. The following table shows the heat transfer coefficient（W·K^-1^·m^-2^） data in Fig. 5.

| flow rate (kg/s) | experiment values | grid numbers 60473 | grid numbers 145643 | grid numbers 365938 |
| --- | --- | --- | --- | --- |
| 0.127 | 1868 | 1777 | 1843 | 1879 |
| 0.155 | 1913 | 2129 | 1862 | 1864 |
| 0.166 | 1951 | 1952 | 1917 | 1878 |
| 0.21 | 2039 | 2408 | 2012 | 2105 |
| 0.264 | 2180 | 2061 | 2112 | 2144 |
| 0.324 | 2290 | 2515 | 2251 | 2339 |

Fig. 5 Test of independence

1. The experimental conditions are shown in Table 1.

Table 1 Experimental condition

| Pressure*10^-5^/Pa | Flow rate/kg·s^-1^ | Gas phase | Heat flux/W·m^-2^ | heat transfer coefficient/W·K^-1^·m^-2^ |
| --- | --- | --- | --- | --- |
| 4.04 | 0.127 | 0 | 3950.99 | 1868.08 |
| 3.98 | 0.155 | 0 | 3950.99 | 1913.31 |
| 4.00 | 0.166 | 0 | 3950.99 | 1951.10 |
| 4.01 | 0.210 | 0 | 3950.99 | 2039.27 |
| 4.01 | 0.264 | 0 | 3950.99 | 2180.20 |
| 3.99 | 0.324 | 0 | 3950.99 | 2290.43 |

1. The following table shows the heat transfer coefficient（W·K^-1^·m^-2^） data in Fig. 6.

| flow rate (kg/s) | simulated values | experimental values | Neeraas model |
| --- | --- | --- | --- |
| 0.127 | 1843 | 1868 | 1884 |
| 0.155 | 1862 | 1913 | 1937 |
| 0.166 | 1917 | 1951 | 1952 |
| 0.21 | 2012 | 2039 | 2002 |
| 0.264 | 2112 | 2180 | 2053 |
| 0.324 | 2251 | 2290 | 2102 |

Fig. 6 Heat transfer coefficient with different mass flow rate inlet

1. The following table shows the heat transfer coefficient（W·K^-1^·m^-2^） data in Fig. 11.

| flow rate (kg/s) | 5° | 8° | 11° |
| --- | --- | --- | --- |
| 0.127 | 1966 | 1843 | 1635 |
| 0.155 | 2044 | 1862 | 1652 |
| 0.166 | 2092 | 1917 | 1654 |
| 0.21 | 2067 | 2012 | 1699 |
| 0.264 | 2186 | 2112 | 1727 |
| 0.324 | 2318 | 2251 | 1833 |

Fig. 11 Heat transfer coefficient varies with mass flow rate of differen winding angle

1. The following table shows the heat transfer coefficient（W·K^-1^·m^-2^） data in Fig. 12.

| flow rate (kg/s) | P_l_=14mm | P_l_=17mm | P_l_=20mm |
| --- | --- | --- | --- |
| 0.127 | 2086.5 | 1843 | 1422 |
| 0.155 | 2145.5 | 1862 | 1494 |
| 0.166 | 2153.7 | 1917 | 1526 |
| 0.21 | 2163.1 | 2012 | 1512 |
| 0.264 | 2242.3 | 2112 | 1787 |
| 0.324 | 2330.7 | 2251 | 1967 |

Fig. 12 Heat transfer coefficient varies with mass flow rate of differen longitudinal distance

1. The following table shows the heat transfer coefficient（W·K^-1^·m^-2^） data in Fig. 13.

| flow rate (kg/s) | d=9 mm | d=12 mm | d=15 mm |
| --- | --- | --- | --- |
| 0.127 | 1421 | 1843 | 1895 |
| 0.155 | 1498 | 1862 | 1990 |
| 0.166 | 1546 | 1917 | 1993 |
| 0.21 | 1580 | 2012 | 2130 |
| 0.264 | 1627 | 2112 | 2823 |
| 0.324 | 1664 | 2251 | 3233 |

Fig. 13 Heat transfer coefficient varies with mass flow rate of differen outside tube diameter

1. The following table shows the heat transfer coefficient（W·K^-1^·m^-2^） data in Fig. 14.

| sloshing amplitude（°） | 5° | 8° | 11° |
| --- | --- | --- | --- |
| 0 | 1966 | 1843 | 1635 |
| 3 | 1979 | 1891 | 1285 |
| 5 | 1879 | 1597 | 1281 |
| 7 | 1582 | 1380 | 1157 |
| 9 | 1431 | 1145 | 1014 |

Fig. 14 Heat transfer coefficient with sloshing amplitude of different winding angle

1. The following table shows the heat transfer coefficient（W·K^-1^·m^-2^） data in Fig. 15.

| sloshing amplitude（°） | P_l_=14mm | P_l_=17mm | P_l_=20mm |
| --- | --- | --- | --- |
| 0 | 2086 | 1843 | 1422 |
| 3 | 2444 | 1891 | 1535 |
| 5 | 2323 | 1597 | 1499 |
| 7 | 2223 | 1380 | 1296 |
| 9 | 1664 | 1145 | 1094 |

Fig. 15 Heat transfer coefficient with sloshing amplitude of different longitudinal distance

1. The following table shows the heat transfer coefficient（W·K^-1^·m^-2^） data in Fig. 16.

| sloshing amplitude（°） | d=9 mm | d=12 mm | d=15 mm |
| --- | --- | --- | --- |
| 0 | 1421 | 1843 | 1895 |
| 3 | 1241 | 1631 | 1746 |
| 5 | 1203 | 1597 | 1700 |
| 7 | 1186 | 1380 | 1408 |
| 9 | 1099 | 1145 | 1345 |

Fig. 16 Heat transfer coefficient with sloshing amplitude of different ouside tube diameter

1. The following table shows the heat transfer coefficient（W·K^-1^·m^-2^） data in Fig. 17.

| sloshing period（s） | 5° | 8° | 11° |
| --- | --- | --- | --- |
| 0 | 1967 | 1843 | 1635 |
| 6 | 1814 | 1502 | 1197 |
| 10 | 1879 | 1597 | 1281 |
| 15 | 2195 | 1679 | 1473 |
| 20 | 2738 | 1807 | 1618 |

Fig. 17 Heat transfer coefficient with sloshing period of different winding angle

1. The following table shows the heat transfer coefficient（W·K^-1^·m^-2^） data in Fig. 18.

| sloshing period（s） | P_l_=14mm | P_l_=17mm | P_l_=20mm |
| --- | --- | --- | --- |
| 0 | 2087 | 1843 | 1422 |
| 6 | 1712 | 1502 | 1342 |
| 10 | 1779 | 1597 | 1381 |
| 15 | 1864 | 1678 | 1514 |
| 20 | 1897 | 1807 | 1556 |

Fig. 18 Heat transfer coefficient with sloshing period of different longitudinal distance

1. The following table shows the heat transfer coefficient（W·K^-1^·m^-2^） data in Fig. 19.

| sloshing period（s） | d=9 mm | d=12 mm | d=15 mm |
| --- | --- | --- | --- |
| 0 | 1421.4 | 1843 | 1894.63 |
| 6 | 1032.5873 | 1502.15134 | 1529.45045 |
| 10 | 1203.1637 | 1596.95624 | 1699.7704 |
| 15 | 1293.73471 | 1678.77959 | 1856.9828 |
| 20 | 1309.10965 | 1806.71529 | 1950.05903 |

Fig. 19 Heat transfer coefficient with sloshing period of different ouside tube diameter
